# Augmenting the Cytotoxicity of Anticancer Peptide K6L9 by In Vitro-Synthesized mRNA

**DOI:** 10.3390/app16073288

**Published:** 2026-03-28

**Authors:** Muturi Njoka, Obdulia Covarrubias-Zambrano, Aprajita Tripathi, Nadine Santana-Magal, John Jeppson, David Akhavan, Kalyani Pyaram, Stefan H. Bossmann, Divya Kamath

**Affiliations:** 1 Department of Cancer Biology, University of Kansas Medical Center, Kansas City, KS 66160, USA; 2 Department of Radiation Oncology, University of Kansas Medical Center, Kansas City, KS 66160, USA

**Keywords:** mRNA therapy, anticancer peptides, K6L9, breast cancer cells

## Abstract

Anticancer peptides (ACPs) offer a promising alternative to conventional chemotherapy but face challenges, including poor selectivity, limited tumor penetration, low cellular uptake, and rapid degradation in serum. To address these barriers, we developed synthetic mRNAs encoding chimeric ACPs designed for enhanced intracellular delivery and activity. mRNAs for constructs SAK6L9AS(1X), SAK6L9AS(4X), and WTAS-K6L9(4X) were transcribed in vitro and tested against 4T1 breast cancer cells. Cytotoxicity was assessed by cell confluence and MTT assays, while apoptosis was evaluated using caspase 3/7 activation, PI staining, and Annexin V flow cytometry. Our results demonstrate that all SAK6L9AS variants induced robust apoptosis and cellular toxicity in 4T1 cells. Importantly, this work provides the first demonstration of intracellular expression of an mRNA-encoded ACP fused to a cell-penetrating peptide, thereby validating a modular platform for RNA-based delivery of anticancer agents. This study highlights the feasibility of mRNA-encoded peptide therapeutics as a scalable and customizable strategy for cancer treatment. By combining the advantages of mRNA delivery with rational peptide design, ACP chimeras can be expressed directly inside tumor cells, overcoming the limitations of exogenous peptide administration. Our findings support further development of synthetic mRNA therapeutics to generate potent, selective anticancer peptides with reduced systemic toxicity and improved translational potential.

## Featured Application

RNA therapy has come a long way, not only as a vaccination alternative but also for therapy. The development and manufacturing of RNA therapeutics are relatively simple and more cost-effective compared to those for recombinant proteins or small molecules. Here, we use mRNA to produce chimeric anticancer peptides that show the potential to induce cell death in not only the cancer cell that harbors and produces them, but also the cells around it. Overall, we see that the mRNA translated into protein in the cells has similar effects to the anticancer peptide. The benefit of this method is that the designer synthetic mRNAs can be easily customized to target specific cancer types for better outcomes.

## 1. Introduction

Messenger RNAs (mRNAs) are an essential link connecting genetic information to protein expression in an organism. The ability to synthesize mRNA synthetically in vitro has been a pivotal step in the field of science. Recent advances in designing, modifying and customizing these in vitro-synthesized mRNAs have revolutionized the field of therapeutics and prophylaxis [1–3].

mRNA therapy has an extensive footprint in cancer therapeutics with currently over 100 IVT mRNAs being tested in clinical trials for cancer immunotherapy [4]. Despite all the advances in therapies, cancer remains a leading cause of mortality worldwide [5]. The complexity of the disease and the increasing incidences of drug resistance elevate the need for newer novel therapies that can be used in combination with the standard of care. One such innovative approach that has reemerged is the repurposing of antimicrobial peptides that show activity against cancer cells in some of the existing literature [6–9]. These anticancer peptides (ACPs) are small peptides (5–50 amino acids in length) [10], with either α-helical or β-sheet secondary structures [11]. ACPs are known to preferentially disrupt the mitochondrial membrane of cancer cells (vs normal cells) as they bear resemblance to the bacterial membrane [12]. Giuliani et al. and Torfoss et al. have shown that ACPs are not directed against specific extracellular or intracellular receptors, but do interact selectively with external, internal, and mitochondrial membranes of cancer cells [13,14]. Some ACPs also show cytotoxicity in multidrug-resistant cancer cells [15]. However, ACPs have low selectivity, poor tumor permeability, and limited tumor cell uptake, and are easily inactivated in serum [16]. Tumor permeability also decreases with an increase in peptide size [17]. Various strategies like nanoparticle drug delivery systems, intracellular pathway delivery, and ligand-mediated tumor targeting (ACP chimeras) have been used to address these challenges [12,17]. ACPs have also been conjugated to other peptides to enhance potency and selectivity in both in vitro and in vivo models [9,12]. For example, D-K6L9 (LKLLKKLLKKLLKLL) is a synthetic antimicrobial peptide that is cytotoxic towards cancer cells because they have 3–9% more phosphatidylserine (PS) [18–22] and O-glycosylated mucins [23,24] on their surface, compared to normal cells. The interaction between the anionic cancer cell membrane components and the cationic D-K6L9 peptide rapidly kills the cells [25]. D-K6L9’s broad spectrum of activity shows synergy with classical chemotherapy [26] and prevents metastasis [8]. However, the threshold lytic concentration of D-K6L9 was above 10 μM, which is unacceptable for therapeutic applications. To improve this, our group added serine (S) and alanine (A) to each end of D-K6L9, creating SA-D-K6L9-AS (SA-LKLLKKLLKKLLKLL-AS), which targets the cell membrane and cell organelles, thereby increasing its cytotoxicity. To avoid immune stimulation, the SA-D-K6L9-AS was packed in mesoporous silica (nano)particles and sealed with polysilazane designer copolymers as gatekeepers, and then loaded into murine neural stem cells to target tumors [27].

We propose combining the advances in IVT technology and ACPs to transfect the fast-growing cancer cells to create a system generating cytotoxic peptides. Including WTAS, which is a cell-penetrating peptide our group previously designed by modifying the microtubule-associated sequence (MTAS) peptide, should enhance this activity [28]. Here, we designed, transcribed, and expressed in vitro mRNAs encoding hybrid peptide (chimera) containing a pro-apoptotic peptide and a cell-penetrating peptide. mRNAs for SA-K6L9-AS (SA-LKLLKKLLKKLLKLL-AS) peptide alone or with the fusion peptide WTAS-SA-K6L9-AS were expressed and the cytotoxicity was determined. With recent studies showing renewed interest and effectiveness of ACPs in breast cancer cells [29–31], we used murine 4T1 cells to test mRNA expression of ACPs. To our knowledge, this is the first work to express an ACP cell-penetrating peptide chimera via mRNA in cancer cells.

## 2. Materials and Methods

### 2.1. Cell Culture

The 4T1 murine breast cancer cells (routinely cultured in the Bossmann Lab at KUMC, were a kind gift from Dr. Derly Troyer from Kansas State University) were thawed and maintained in complete media containing Roswell Park Memorial Institute (RPMI) 1640 medium (Sigma-Aldrich, Inc., St. Louis, MO, USA) supplemented with 10% FBS (fetal bovine serum; Sigma-Aldrich, Inc., St. Louis, MO, USA) without antibiotics. Cells were maintained at 37 °C in 5% CO_2_ and humidified conditions (90% relative humidity). The cells were routinely screened for bacteria and spores using an inverted microscope (EVOS M5000, Invitrogen, Washington, DC, USA). Mycoplasma contamination was tested using MycoStrip^®^ (#rep-mys-20, InvivoGen, San Diego, CA, USA) following the manufacturer’s instructions.

### 2.2. Design and Amplification of SA-K6L9-AS DNA Template

The plasmid DNA (pDNA) construct used to synthesize SA-K6L9-AS mRNA was flanked by an EcoR1 restriction site (5′-GAATTC-3′). The 5′ untranslated region (UTR) contained the T7 promoter (5′-TAATACGACTCACTATAGGG-3′) and the Kozak sequence with a start codon (5′-GCCATGGGT-3′). The amino-terminal enhancer of split (AES) motif followed by non-coding mitochondrial 12S rRNA (mtRNR1) motif was used as the 3′UTR. Each SA-K6L9-AS repeat was separated by a linker (5′-GGSGGS-3′) and a high-fidelity hexanucleotide stop codon (5′-AATAAA-3′). The sequences were optimized by GenScript web-based software (GenSmart 2.0) [32] and common restriction enzyme cutting sites were mapped using the NEBcutter v3.0.19 tool (New England Biolabs). The template DNA containing the coding sequences (CDSs) for the mRNA was synthesized and cloned into either the pMA-RQ plasmid or pUC-GW plasmid (Genewiz, South Plainfield, NJ, USA).

The pDNA containing the SA-K6L9-AS template was restriction-digested using the EcoRI enzyme (New England Biolabs, Ipswich, MA, USA) as per the manufacturer’s instructions. Briefly, reaction mixture containing 0.5 μg plasmid DNA, 2 μL 10X NEBuffer, 7 μL nuclease-free water, and 1 μL EcoRI enzyme was incubated at 37 °C for 2 h. The digested plasmid was purified using Monarch PCR & DNA Cleanup Kit (New England Biolabs, Ipswich, MA, USA). Restriction digestion was confirmed by resolving 2 μL of the reaction mixture on 1% agarose gel. The DNA inserts and pMA-RQ/pUC-GW vector backbone bands were identified for the correct sizes. The sequence of interest was amplified using the Platinum^™^ PCR SuperMix High Fidelity kit (Life Science Technologies, Carlsbad, CA, USA) used per the manufacturer’s instructions. The DNA concentration was determined using a NanoDrop OneC spectrophotometer (Thermo Scientific, Wilmington, DE, USA), and its preliminary quality, size, and purity were checked using 1% agarose gel electrophoresis. Finally, the DNA template (PCR product) was validated through Sanger sequencing [33] at Azenta Life Sciences (Genewiz, South Plainfield, NJ, USA).

### 2.3. In Vitro Transcription (IVT) of mRNAs

mRNA was synthesized using the HiScribe T7 ARCA mRNA Kit (with capping and tailing, New England Biolabs, Ipswich, MA, USA) following the manufacturer’s protocol. Briefly, the IVT reaction mixture contained 10 μL 2X ARCA/NTP mix, 6 μL of nuclease-free water, 1 μg of template DNA, and 2 μL of T7 RNA polymerase mix. This mixture was mixed and incubated at 37 °C for 45 min, after which 2 μL of DNase I was added to the reaction and incubated at 37 °C for 15 min. To add Poly(A) tail, 5 μL of Poly(A) buffer, 5 μL of *E. coli* Poly(A)A polymerase, and 20 μL of nuclease-free water were added to the IVT reaction mix and incubated at 37 °C for 30 min. The mRNA was purified using a Monarch RNA cleanup kit (New England Biolabs, Ipswich, MA, USA) and eluted twice in 20 μL of nuclease-free water. The mRNA concentration was determined using a NanoDrop OneC spectrophotometer (Thermo Fisher Scientific, Madison WI, USA), and the purity, size, and quality were confirmed using agarose gel (1%) electrophoresis. The IVT mRNA was aliquoted and stored at −80 °C for transfection experiments.

### 2.4. Assessment of IVT mRNAs Using RNA ScreenTape Assay

The Agilent TapeStation 4200 System (Agilent, Santa Clara, CA, USA) was used to assess the size and purity of IVT mRNAs. The mRNAs were diluted 10X with nuclease-free water (~50 ng/μL), heated at 72 °C for 3 min to denature, and snap-cooled on ice for 1 min. The mRNAs were loaded into the Agilent RNA ScreenTape using Agilent RNA ScreenTape Sample Buffer and resolved by capillary electrophoresis. Also, the Agilent RNA ScreenTape Ladder (size standard) was run in parallel.

### 2.5. Transfecting Cells with mRNAs

In vitro transfection of SA-K6L9-AS (hereby referred to as K6L9 instead of SA-K6L9-AS) IVT mRNAs was carried out with Lipofectamine 2000 (Life Science Technologies, Carlsbad, CA, USA) according to the manufacturer’s protocol. Experiments were performed with 4T1 cells (4 × 10^4^ cells per well) in 96-well plates. The cells were seeded for 24 h to ~20% confluency before transfection. For transfection, 25 μL of reduced serum medium (Opti-MEM, Life technologies corporation, Grand Island, NY, USA), 0.3 μL of Lipofectamine 2000, and 0.4 to 2 pmol SA-K6L9-AS IVT mRNAs were mixed and incubated for 5 min at room temperature to form lipoplexes. Culture media were aspirated from seeded cells and incubated with lipoplexes for 24 to 48 h post-transfection at 37 °C in 5% CO_2_ in an IncuCyte S3 live-cell analysis system (Sartorius AG, Göttingen, Germany). The cell images were scanned at regular intervals using a 10X objective lens for the duration of the experiment. The percentage confluency and total fluorescence counts were calculated from the images acquired using the IncuCyte software v2023A.

### 2.6. MTT Cell Viability Assay

An MTT (3-(4,5-dimethylthiazol-2-yl)-2,5-diphenyltetrazolium bromide; Sigma-Aldrich, Inc., St. Louis, MO, USA) assay [34] was used to assess the cytotoxic effect of IVT-generated mRNA 24 and 48 h post-transfection. Twenty microliters of MTT solution (5 mg mL^−1^) were added to each well and incubated for 4 h. Following the incubation, the media were removed, and the formazan crystals were dissolved in 100 μL of DMSO (dimethyl sulfoxide) with shaking for 5 min. The optical density was recorded using a microplate reader (SynergyH1 BioTek, Winooski, VT, USA) at 550 nm for test and 690 nm for reference. The inhibition and viability data were plotted as percent change in cell viability compared to the vehicle control.

### 2.7. Apoptosis Analysis by IncuCyte Live-Cell Analysis System

Experiments were performed with 4T1 cells (4 × 10^4^ cells per well) in 96-well plates. The cells were seeded for 24 h to ~20% confluency and transfected with K6L9 IVT mRNAs. The IncuCyte live-cell analysis system was used, which provides kinetic data and can be combined with fluorescent data to give more information than a snapshot endpoint readout. Apoptosis was evaluated by incubating cells with membrane-permeable IncuCyte caspase-3/7 green dye (5 μM, 1:1000 dilution), IncuCyte Annexin V Orange Dye (1:200 dilution), and IncuCyte Cytotox NIR Dye (0.6 μM, 1:1000 dilution, Sartorius, Ann Arbor, Mi, USA). The IncuCyte caspase-3/7 green dye is cell-permeable and intercalates into the DNA after cleavage by caspase-3/7 from apoptosis activity. The IncuCyte Annexin V Orange Dye binds to the phosphatidylserine (PS) exposed to the extracellular membranes of cells undergoing apoptosis. IncuCyte Cytotox NIR Dye binds to cellular DNA when the unhealthy cells lose their plasma membrane integrity, yielding increased fluorescence. Image scheduling and collection were performed with the IncuCyte S3 Live-Cell Analysis System hourly for 24 or 48 h with a 10× objective lens. At each time point, four images were taken per well in brightfield, FITC, orange, and NIR channels. Data collection was set as follows: Segmentation: ‘AI confluence’, Filters: Area (μm^2^) min ‘1000’, and Threshold (G/O/NCU): ‘2’. Spectral unmixing was achieved by subtracting 10% NIR contribution from orange, 2% NIR contribution from green, and 7% orange contribution from green. Finally, the integrated IncuCyte S3 software version 2023A was used to analyze the AI confluence and the fluorescence count in the 4T1 cells.

### 2.8. Apoptosis Analysis by Flow Cytometry

The BD Pharmingen^™^ FITC Annexin V Apoptosis Detection Kit I (BD, Biosciences, Franklin Lakes, NJ, USA) was used to assess cell apoptosis using flow cytometry. The 4T1 cells (2.5 × 10^5^ cells per well) were seeded in 24-well plates for 24 h and transfected with SA-K6L9-AS mRNAs for 24 or 48 h. The cells were washed twice with PBS (with no Ca^2+^/Mg^2+^) and then dissociated using 250 μL Accutase (Gibco/Invitrogen, Merelbeke, Belgium). The cells were again washed twice with PBS and centrifuged at 150× *g* for 5 min. One hundred microliters of 10-fold-diluted Annexin V Binding Solution was used to suspend ~2 × 10^4^ cells. The cells were stained with 1 μL of Annexin V-FITC Conjugate for 15 min in the dark at RT. One microliter of PI Solution was diluted in 400 μL 1× Annexin V Binding Solution, and 10,000–20,000 events per sample were analyzed using the Cytek^®^ Aurora flow cytometry system (Cytek^®^ Biosciences, Bethesda, MD, USA). The flow cytometry data were analyzed using FlowJo software v10.8.1 (Becton, Dickinson and Company (BD, Franklin Lakes, NJ, USA)).

### 2.9. Statistical Analysis

The data were collected in three technical replicates and three experimental replicates per experiment for confluence, MTT, and IncuCyte live imaging. For the flow cytometry experiments, data were collected for 3 experimental replicates. All data are shown as mean ± standard deviation (SD). One-way analysis of variance (ANOVA) with Bonferroni’s post-test was used to compare the differences among multiple groups. The Kruskal–Wallis test was used to compare the tests and their respective controls. Student’s t-test was used to compare the difference between two groups. All the data were analyzed and graphed using GraphPad Prism Software v9.4.1 (La Jolla, CA, USA). The difference was considered statistically significant if *p*-value < 0.05.

## 3. Results

### 3.1. Design, In Vitro Synthesis, and Validation of mRNAs Encoding Chimeric Synthetic Anticancer K6L9 Peptides

Four mRNAs of interest (K6L9 [1X], K6L9 [4X], WTAS-K6L9 [4X] and WTAS-K6L9 [4X]-GFP) were designed, synthesized, and tested in this work. The WTAS-K6L9-GFP was used as a test mRNA to visually validate the translation of mRNA into a protein. The CDSs of the template DNA sequences for the mRNAs (Supplementary Table S1) were cloned into either pMA-RQ or pUC-GW plasmids along with the 5′ and 3′ UTR. The purified plasmids were linearized using EcoRI restriction enzyme digestion and validated for the size of the insert on an agarose gel (Figure 1a; Supplementary Figure S1a, for WTAS-K6L9-GFP). The purified inserts for each of the mRNAs were PCR-amplified, purified, and then used as a template for the in vitro synthesis of respective mRNAs (Figure 1b; Supplementary Figure S1b, for WTAS-K6L9-GFP). The mRNAs were ARCA-capped simultaneously during transcription and were sequentially polyA-tailed. The final capped and tailed mRNA products were tested for their size and purity on the RNA ScreenTape assay and showed clear single mRNA bands with an increase in final product sizes (nt) due to addition of the polyA tail (Figure 1c). WTAS-K6L9(4X)-GFP mRNAs showed two bands (Supplementary Figure S1c) with the higher-molecular-weight band coinciding with the desired size of 1731 nt. The lower-molecular-weight band may have been caused by the inefficiency of the polymerase enzyme in synthesizing longer mRNA (Supplementary Figure S1c). The size and purity of the synthesized mRNAs were confirmed by RNA ScreenTape fragment sizing. The confirmed mRNA sizes had at least 41 polyA bases added (Figure 1d–f; Supplementary Figure S1d). Since the mRNAs were synthesized via in vitro transcription, they lacked ribosomal RNA used as a reference for integrity determination in the RNA ScreenTape assay. To validate the mRNA sequences, we sequenced cDNA, and there was no anomalous base change.

### 3.2. Translation of Anticancer K6L9 Peptide mRNAs Reduced Cellular Proliferation and Induced Cytotoxicity in 4T1 Cells

Lipofectamine 2000 was used as a carrier (vehicle) for the delivery of negatively charged mRNAs so that they were not repelled by the negative charge of the cell’s plasma membrane. The lipofectamine concentration was determined by running a dose curve in the 4T1 cell line and the nontoxic dose of 0.3 μL (as recommended by the manufacturer) was selected for further experimentation. The mRNA–lipofectamine ratio was maintained at 1:3 during the transfection experiments. Converting exogenous mRNA into proteins is a multi-step process and involves the stability of the mRNA inside the vehicle, its endosomal release, and then finally the unpacking and release of the mRNA into the cytoplasm for translation [35–37].

To validate the translation of the mRNAs, 4T1 cells were initially transfected with 2 pmol/well WTAS-K6L9(4X)-GFP mRNA 12 h post-seeding and monitored for a total of 48 h (including the 12 h before transfection) in Sartorius IncuCyte^®^. WTAS-K6L9(4X)-GFP mRNA was used as a control because it encodes the cell-penetrating (WTAS) peptide, cytotoxic (K6L9) peptide, and GFP. Initiation of translation for GFP expression was recorded as early as 6 h post-transfection, with a maximum expression observed between 16 and 22 h post-transfection (Supplementary Figure S2a,b and Video S1). The GFP fluorescence was sustained for approximately 36 h post-transfection. This data provides evidence that the mRNA design supports translation in 4T1 cells. To test the effect of the mRNAs on growth, the 4T1 cells were transfected with three doses of mRNA (0.4, 1.2, and 2 pmol/well; in 96-well plates), maintaining the mRNA–lipofectamine ratio at 1:3. The effect on growth was measured as the change in confluence (percentage area covered by the cells) using IncuCyte^®^ at 24 and 48 h post-transfection. The change in cell confluence was determined as a percentage change compared to lipofectamine control. The effect of the mRNA on cell viability was recorded as percentage viability using the MTT assay and the change in the cell viability over the incubation time was determined as a percent change compared to lipofectamine control.

At 24 h post-transfection, all three mRNAs showed significant reduction in cell confluence and cell viability at 1.2 and 2 pmol RNA dose in the 4T1 cell line. Similar effects were observed at the 48 h time point for K6L9(4X) and WTAS-K6L9(4X) (Figure 2a–f). K6L9(1X) mRNA showed significant reduction in confluence at only the highest concentration of 2 pmol at 48 h, but cell viability was significantly reduced at 1.2pmol and 2 pmol doses (Figure 2a,b). Although there was a trend of reduction in confluence and viability at 0.4pmol dose, it was not statistically significant (Figure 2a–f) for all three mRNAs tested. The WTAS-K6L9(4X)-GFP mRNA showed a significant reduction in cell confluence even at 0.4 pmol concentration at 24 h (Supplementary Figure S2c), and at 48 h significant reduction was observed at 1.2 pmol and 2 pmol (Supplementary Figure S2c). WTAS-K6L9(4X)-GFP mRNA showed reduction in cell viability at 1.2 pmol and 2 pmol at both 24 and 48 h (Supplementary Figure S1d).

### 3.3. The Expression of Synthetic Anticancer K6L9 Peptide mRNAs Activated the Apoptosis Pathway in 4T1 Cells

Apoptosis is a complex pathway initiated by the activation of caspases and ending with cell death. Activation of caspases 3 and 7, the primary executioners, is an irreversible step in the initiation of apoptosis and is considered a marker for the detection of apoptosis in the cell. This is followed by externalization of the phosphatidyl serine (PS) on the plasma membrane and consecutive disruption of the cell membrane permeability. Here, we transfected the 4T1 cells with the mRNA liposomes at three different doses and added caspase 3/7 cleavable DNA dye (green) as a detector for early apoptosis, Annexin V dye (orange) as a detector of changes in externalization of phosphatidyl serine on the plasma membrane, and a plasma-membrane-impermeable dye (NIR) to detect the cell membrane disruption. The cells were monitored, and images were taken at 24 and 48 h. Figure 3a shows the representative images of the caspase dye at 48 h for K6L9(4X) mRNA.

The fluorescence counts from images of each of the dyes were normalized to the vehicle control. At 24 h post-transfection, K6L9(1X) mRNA showed significant caspase activation only at a 2 pmol concentration, while K6L9(4X) and WTAS-K6L9(4X) mRNAs showed significant caspase activation at 1.2 and 2 pmol concentrations (Figure 3b–d). At the 48 h time point, the K6L9(1X) and K6L9(4X) mRNAs showed significant caspase activation at 1.2 and 2 pmol concentration, and the WTAS-K6L9(4X) mRNA showed caspase activation at 2 pmol concentration (Figure 3b–d). However, the 0.4 pmol mRNA concentration had no significant caspase activation in any mRNA. (Figure 3b–d). Significant Annexin V dye binding to externalized PS was detected for K6L9(1X) mRNA at 2 pmol concentration at 24 and 48 h (Supplementary Figure S3a) and WTAS-K6L9(4X)-GFP mRNA at 2 pmol concentration at 48 h (Supplementary Figure S2e,f). There was no significant Annexin V binding for K6L9(4X) or WTAS-K6L9(4X) mRNAs at either of the time points (Supplementary Figure S3c,e). Similarly, there was no significant cell plasma membrane disruption in any of the mRNAs tested (Supplementary Figure S2g for WTAS K6L9(4X) GFP; Figure 3b,d). These data indicate that the translation of synthetic anticancer K6L9 peptide mRNAs activated apoptosis cell death at the tested time points of 24 and 48 h post-transfection. Only K6L9(1X) peptide showed evidence of externalization of the PS membrane at 2 pmol, and none of the peptides showed evidence of membrane permeabilization as determined by the NIR dye.

### 3.4. Translation of Synthetic Anticancer K6L9 Peptides Induced Cellular Toxicity at 48 h in 4T1 Cells

The induction of apoptosis and cell death by cytotoxic mRNA was further validated by flow cytometry using Annexin V and PI dyes, respectively. PI stains only dead cells, and Annexin V stains early-apoptotic cells. The presence or absence of these dyes informs us of the percentage of cells that are affected by the translated peptides. At 24 h post-transfection, the percentage of PI-positive cells in all the mRNA-translated groups was comparable to that in lipofectamine controls as seen in the histograms (Figure 4b,d,f) and the plots (Figure 4a,c,e). No difference was observed in Annexin V at 24 h (Supplementary Figure S4a–f). At 48 h post-transfection, there was a substantial increase in the number of PI-positive cells as seen in the histograms and the plots (Figure 4a–f). We see similar trends for Annexin V (Supplementary Figure S4a–f), indicating that the ACPs generated via the mRNAs exerted a cytotoxic effect on the cells.

### 3.5. ACPs Generated Through mRNA Transfection Are Similar in Cytotoxic Activity

In this study, the three mRNAs were designed to generate the SA-K6L9-AS peptide with different structural features. Traditionally, GGS repeats are utilized as flexible linkers due to their high degree of conformational freedom. However, the SA-K6L9-AS mRNA with 1X shows the formation of a supramolecular structure of GGS repeats, while the K6L9 block appears as a disordered loop (Figure 5a). The SA-K6L9-AS with 4X repeat was designed with GGS linker sequences separating each K6L9 repeat to give it flexibility. However, the structure shows again the presence of a supramolecular structure of GGS repeats and disordered K6L9 loops. The third mRNA design included a cell penetration ligand, WTAS, to confer the ability of transfecting additional cells that may not have taken up the mRNA and to give a cytotoxic advantage to our fusion WTAS-K6L9(4X) peptide over the other two mRNA designs (Figure 5c). All three mRNAs were found to be effective as shown in the previous figures. However, to investigate if one mRNA design resulted in a peptide with better activity than the others, we reanalyzed the existing data and compared them head to head for confluence, viability, and caspase activation.

The three peptides showed comparable effects at 2 pmol and 1.2 pmol doses at both 24 h and 48 h for change in confluence. However, at 0.4 pmol K6L9(4X) and WTASK6L9(4X)-GFP showed better reduction in confluence than K6L9(1X) at 24 h, though the effect was comparable at 48 h (Figure 5d,f,h) across the three mRNAs. For the cell viability assay, K6L9(4X) showed higher reduction in cellular viability at 2 pmol at 24 h but not at 48 h, potentially due to loss of cells at 24 h. At 0.4 pmol K6L9(4X) showed higher reduction compared to the other two mRNAs at 48 h whereas similar activity was observed at 24 h between the three peptides. At 1.2 pmol all the peptides showed similar activity across the parameters tested. For the caspase activation using the IncuCyte dyes, all three peptides showed similar activity at 0.4 pmol (24 h and 48 h) and 1.2 pmol (24 h). K6L9(4X) showed lower activity at 1.2 pmol (24 h) and 2 pmol (24 and 48 h) compared to the other two peptides (Figure 5j–l). Similar activities were also observed for Annexin V and NIR dyes (Supplementary Figure S5a–f).

## 4. Discussion

Here, we demonstrated the feasibility of generating cytotoxic anticancer peptides (ACPs) using in vitro-transcribed (IVT) mRNAs in a murine breast cancer cell line. We designed and synthesized mRNAs of a known anticancer peptide, SA-K6L9-AS, and its chimera/fusion peptides to test the effect of its translation on the cancer cell line. The translated mRNAs reduced cell confluence and viability by apoptosis as determined by the MTT assay and caspases 3/7 activation. A dose-dependent response was observed with significant cytotoxic effect at higher concentration of the mRNAs, with minimal effects observed at the lowest tested dose of 0.4 pmol. Overall, ACPs, when introduced intracellularly as mRNAs in murine breast cancer cells, have a similar cytotoxic effect on the cells to that observed when the cells are treated with the peptide. Although there are differences in kinetics of translation and post-transcriptional regulations in human and murine cells, the IVT mRNA technology has been previously shown to be successful in both human and murine cells in the literature. In this work, we show the activity of IVT mRNA for ACPs in murine cell lines. Further studies are needed to confirm these findings in human cell lines.

With advances in mRNA technology, it is possible to design the coding sequences to address the primary goal of the project. The focus of the mRNA design for this project involved high stability and translational efficiency. This would ensure that the once the mRNA is released into the cytoplasm of the cells after endocytosis, the dose of mRNA given would yield multiple copies of the polypeptide. For this we used the amino-terminal enhancer of split (AES) motif followed by a non-coding mitochondrial 12S rRNA (mtRNR1) motif as the 3′ untranslated regions (UTRs; Supplementary Table S1), which were optimized for translation efficiency [38–40]. The intracellular stability of the mRNAs was increased by adding a 5′-cap structure [41–44] and 3′ polyA tail [39,45,46].

Using GFP-tagged WTAS-SA-K6L9-AS as a control, we determined the feasibility of using IVT-generated mRNA for translation into the protein. Translation of mRNA as determined by the expression of the GFP protein was observed at ~6 h and was sustained for ~36 h post-transfection. We observed peak GFP signal 16–22 h post-transfection, potentially indicating generation of multiple copies of the polypeptide from the transfected mRNA. This alludes to the stability of the exogenous mRNA as well as ability of the cells to synthesize and accept production of anticancer peptide. Any GFP expression earlier than 2 h or later than 36 h post-transfection was not captured due to our experimental design.

Apoptosis is a programmed cell death mechanism that is initiated with the activation of the caspases, followed by changes in the plasma membrane, nuclear condensation, DNA fragmentation, and cell death via shrinkage. It is also characterized by loss of cell membrane integrity [47]. Using dyes that can be incubated at 0 h post-transfection, we monitored the effect of the mRNAs for apoptosis by tracking caspases, Annexin V, and NIR. We observed a significant increase in caspase activation at higher doses, whereas at lower concentrations it was more similar to the lipofectamine control. This aligns with our confluence and viability data. Although we saw a similar trend at 48 h, the fluorescence values were lower for caspase activation, probably due to the cytotoxic effect of the peptides, reducing the number of cells in the well.

Annexin V was used as a detector of changes in externalization of phosphatidyl serine (PS) of the plasma membrane and NIR was used to determine the integrity of the plasma membrane. Although we saw a robust significant signal for activation of apoptosis via caspases, we did not see significant changes in Annexin V or NIR in any of the mRNA used except in K6L9(1X), which showed significant Annexin V activity at both 24 and 48 h. However, we did see a trend towards a slight increase in the signal, but it was not significant. This could indicate the initiation of apoptosis but no membrane damage at least until 48 h, potentially due to the interaction of the ACP with the plasma membrane hindering its detection in the method used in this work. The peptides probably bind to the plasma membrane, preventing the membrane flipping, as well as membrane permeability confounding the data obtained using Annexin V and NIR. Although robust experimentation is required to further confirm this hypothesis, preliminary evidence from the GFP ACP video (Supplementary Video S1) shows that there is an increase in GFP signal over a period of 48 h as the cell replicates, but we did not see the GFP-peptide being released/excreted out of the cells.

The mRNAs were designed such that the 4X repeats and WTAS(4X) would have an advantage over the 1X repeat in terms of increased cytotoxicity. Comparing the data head to head shows some differences but without a consistent pattern. Based on the structures of the peptides (Figure 5) derived using SWISS-MODEL, it is possible that the SGGSGGSGGSGGS sequences that were integrated to permit mobility of individual K6L9 units with respect to each other formed supramolecular aggregates driven by hydrogen bonding between serine -OH groups and carbonyl groups of the backbone. Additional hydrogen bonds were formed between the backbone amide groups and backbone carbonyl groups. PDB files of all four peptides are provided in the Supplementary Information section. This supramolecular structure formation had the consequence that the ALKLLKKLLKKLLKLLA sequences formed loops. To have an advantage, the K6L9 mRNA design needs to be further modified and tested in subsequent studies.

K6L9 peptide and its derivatives have been studied widely. These peptides are amphipathic because of the repetition of hydrophobic leucine and hydrophilic lysine units. Due to their positive net charges under physiological conditions, K6L9-type peptides are electrostatically attracted to negatively charged acidic phospholipids, such as phosphatidyl serine, which are often enriched in the membranes of cancer cells [48]. Membrane binding changes the secondary structure of the peptide to predominantly distorted/dynamic helix, which permits membrane insertion, followed by membrane destabilization. Although the initial binding between K6L9 and the membrane is caused by electrostatic attraction, the observed membrane destabilization is triggered by hydrophobic interactions [25]. It should be mentioned that replacing some L-amino acids of K6L9 with D-amino acids enhances the lytic abilities of these small peptides. However, this strategy is not possible when delivering mRNA. Since most of the available data on using K6L9 and its derivatives are based on delivering the peptide, the mechanism of using mRNA to synthesize the peptide in the cells is novel and needs to be studied more.

Overall, we see the mRNA translated into the protein inside the cells having a cytotoxic effect, as would have been seen when using a peptide. This is one of the first studies to our knowledge that uses mRNA to generate ACPs in cancer cells and uses CPP to enhance the reach of the ACPs. Further studies are needed to modify the sequence of the mRNA to generate a peptide that will be released from cells and also understand the mechanism of action. More work is also needed in developing ACP mRNA therapeutics to circumvent ACPs’ in vivo toxicity and improve delivery specificity. The mRNA technology is versatile. Research on different formulations of synthetic mRNAs with modified coding/non-coding elements and nanoparticle delivery systems will improve mRNA targeting and expression duration.

The feasibility of using mRNA to generate ACPs sets the foundations for further studies that improve the specificity of such peptides. A comparative study of peptide and its modifications synthesized through solid-state synthesis and mRNA translation will give further insights into the benefits of using this method. Such studies will also help understand the kinetics, stability and activity of the peptides generated through these methods. Combining these methods with specific delivery mechanisms can further advance targeted cancer treatments reducing non-specific treatment issues.

## 5. Conclusions

Here, we designed, transcribed, and expressed in vitro mRNAs encoding K6L9 (SALKLLKKLLKKLLKLLAS) peptides K6L9(1X) and K6L9(4X) and a fusion peptide, WTAS-K6L9(4X) and tested their effects on a murine breast cancer cell line. We observed toxicity from the translated mRNA as observed in ACPs synthesized as peptides. To our knowledge, this is the first report of an mRNA encoding an ACP cell-penetrating peptide chimera that targets cancer cells. The mRNA anticancer therapeutic strategy demonstrated herein not only permits individualized and modular cancer treatment regimens but also provides a means of circumventing the cytotoxicity and off-target effects associated with ACP therapy.

## Supplementary Material

applsci-4165037-Video S1

applsci-4165037-supplementary tables and figures_A

**Supplementary Materials:** The following supporting information can be downloaded at https://www.mdpi.com/article/10.3390/app16073288/s1. Figure S1: In vitro synthesis and validation of mRNAs; Figure S2: Expression of WTAS-K6L9(4X)-GFP mRNA in 4T1 cells induces cytotoxicity; Figure S3: Translation of K6L9 mRNAs had no significant effects in Annexin V binding or cell membrane integrity at 24 and 48 h post-transfection; Figure S4: Cytotoxic effect of the translated peptide observed at 48hrs; Figure S5: ACPs generated through mRNA transfection are similar in cytotoxic effect; Figure S6: Representative flow cytometry 4-quadrant graphs for ACPs generated through mRNA transfection. Table S1: DNA template sequences for K6L9(1X), K6L9(4X, WTAS-K6L9(4X) and WTAS-K6L9(4X)-GFP mRNA, AES-3′UTR, and mtRNR1–3′UTR; Video S1: Time lapse video of GFP expression in 4T1 cells transfected with GFP-WTAS-K6L9(4X).

## Figures and Tables

**Figure 1.**
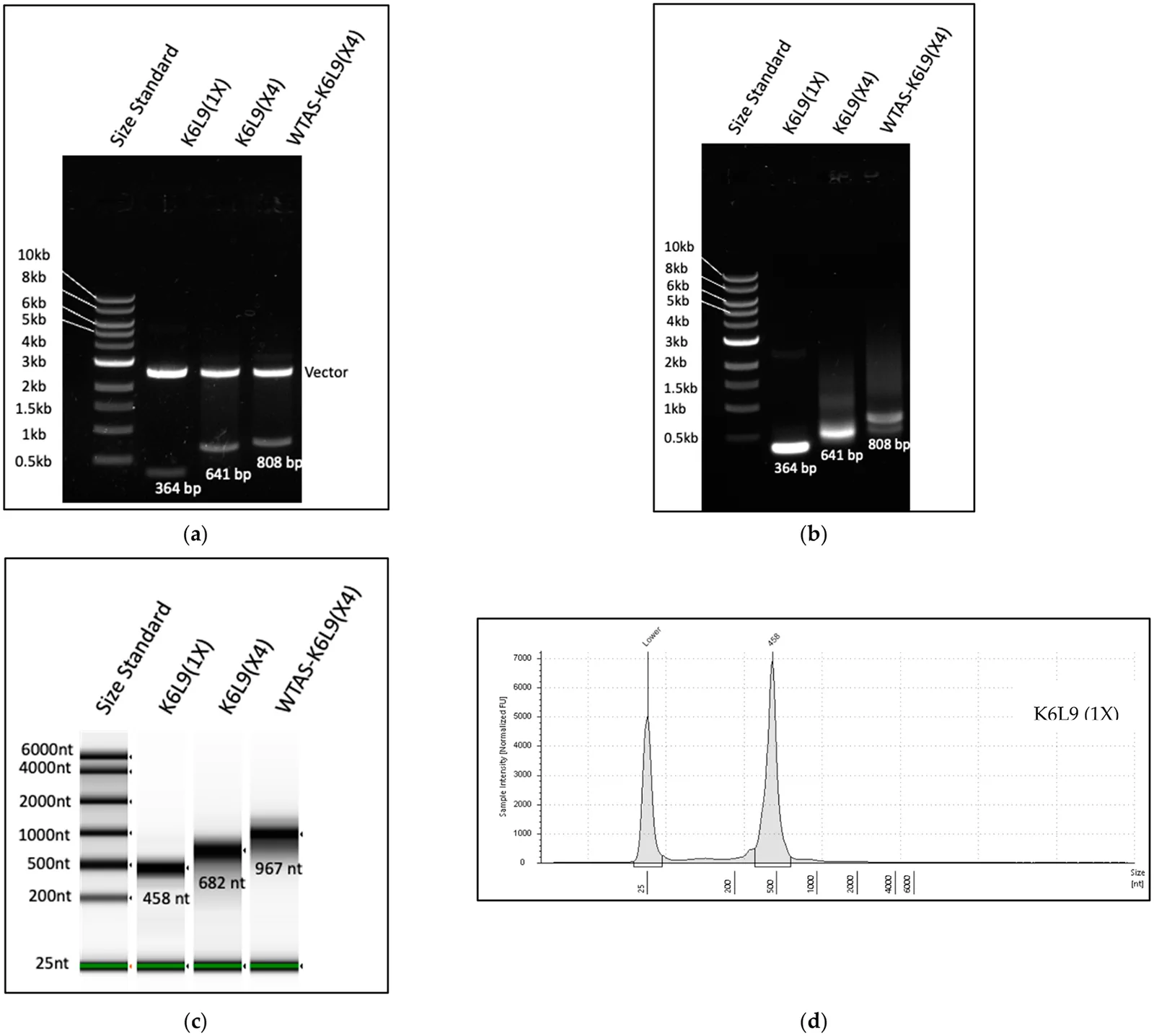

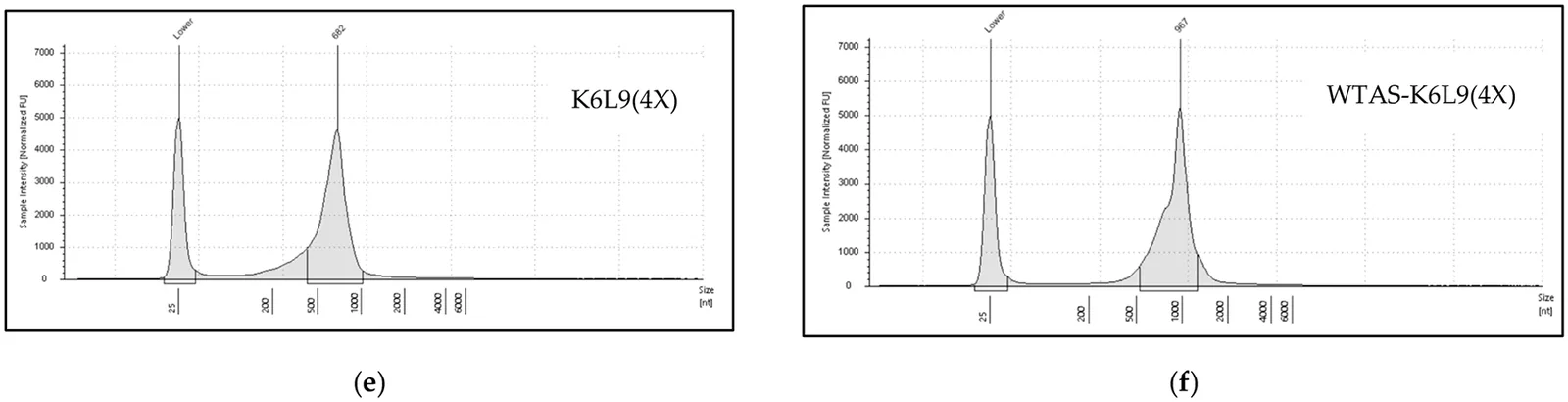
In vitro synthesis and validation of IVT-generated mRNAs. (**a**) Agarose gel image showing restriction digestion of the cloning plasmids carrying K6L9(1X), K6L9(4X) and WTAS-K6L9(4X) template DNA inserts. Size standard is indicated in kilobase pairs (kb), while band sizes are indicated in base pair (bp). Vector refers to the plasmid backbone. (**b**) Agarose gel image showing PCR-amplified K6L9(1X), K6L9(4X) and WTAS-K6L9(4X) template DNA. Size standard is indicated in kilobase pairs (kb), while band sizes are indicated in base pair (bp). (**c**) RNA ScreenTape image showing IVT-synthesized mRNA bands of K6L9(1X), K6L9(4X), and WTAS-K6L9(4X). Size standard and band sizes are indicated in nucleotides (nt). (**d**) RNA ScreenTape assay electropherogram showing the K6L9(1X) mRNA size in nt and peak height as normalized intensity. (**e**) RNA ScreenTape assay electropherogram showing the K6L9(4X) mRNA size in nt and peak height as normalized intensity. (**f**) RNA ScreenTape assay electropherogram showing the WTAS-K6L9(4X) mRNA size in nt and peak height as normalized intensity.

**Figure 2.**
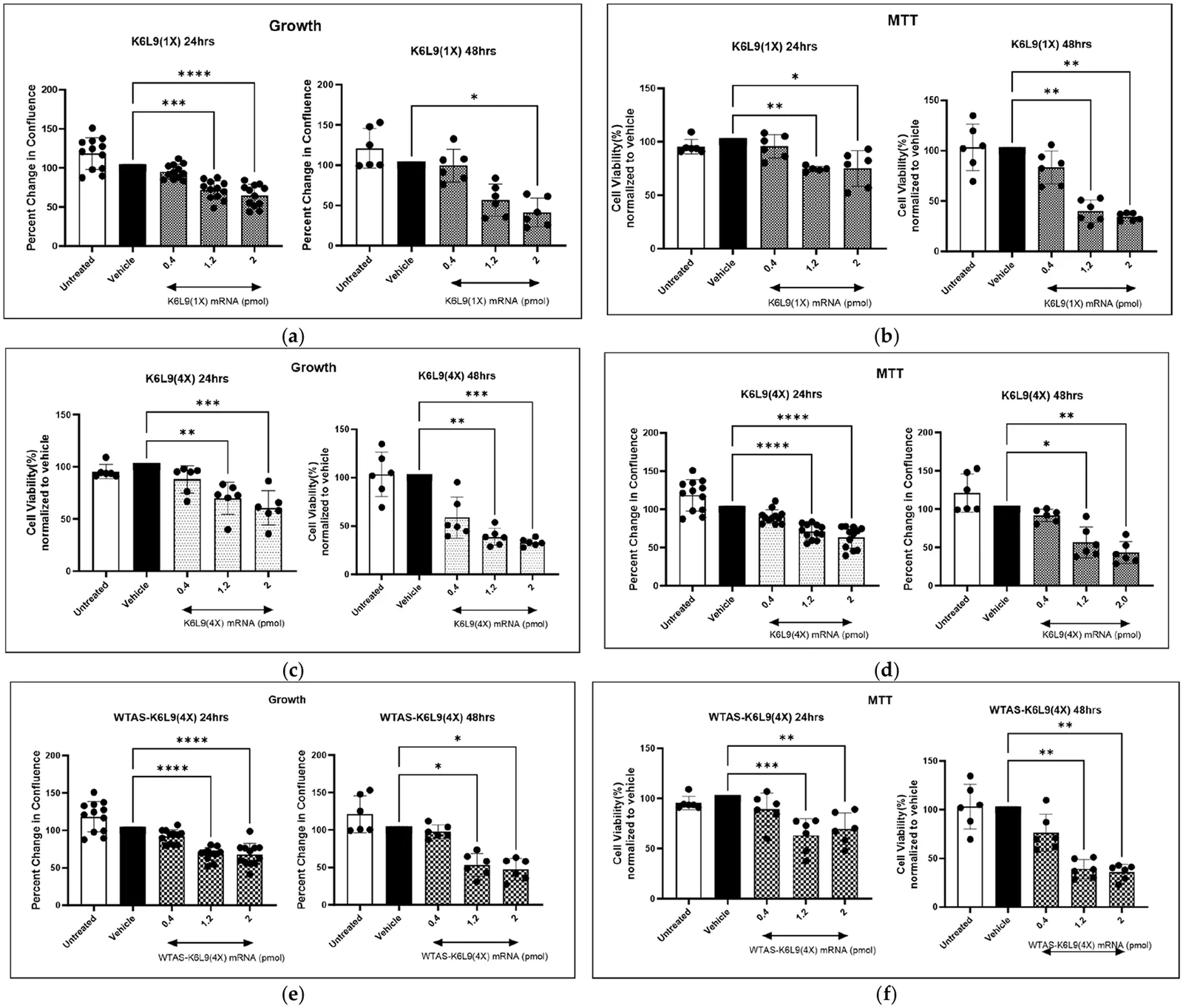
Expression of K6L9(1X), K6L9(4X), and WTAS-K6L9(4X) mRNAs reduced 4T1 cell proliferation and viability in vitro. 4T1 cells were transfected with mRNA at 0.4, 1.2, and 2 pmol and monitored for change in cell growth for 24 and 48 h. Cell growth was measured as percent total area covered by cells (confluence). Confluence was normalized and plotted as a percentage of the vehicle. Data are represented as change in confluence for (**a**) K6L9(1X), (**c**) K6L9(4X), and (**e**) WTAS-K6L9(4X). Cell viability is determined by MTT assay at 24 and 48 h after transfection of the mRNAs. Data are represented as percent viability normalized to the vehicle for (**b**) K6L9(1X), (**d**) K6L9(4X), and (**f**) WTAS-K6L9(4X). The data were collected from three technical and experimental replicates and recorded as mean ± SD. * = *p* < 0.05, ** = *p* < 0.01, *** = *p* < 0.001, **** = *p* < 0.0001.

**Figure 3.**
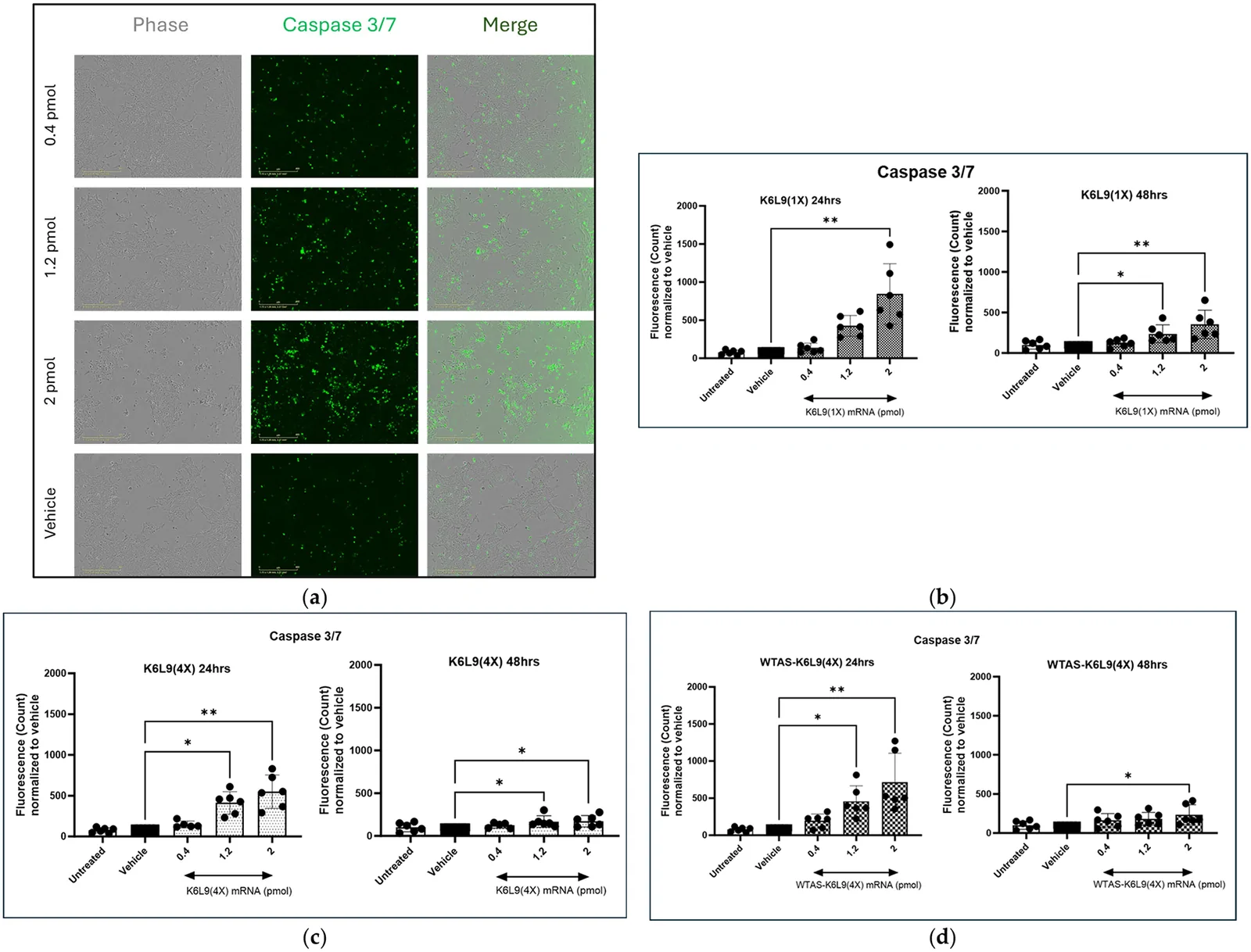
Translation of K6L9 cytotoxic mRNAs induces apoptosis in 4T1 cells. (**a**) Representative image showing caspase 3/7 activation after transfecting the 4T1 cells with K6L9(4X) mRNA at 48 h. Caspase 3/7 activity is measured by fluorescence counts emitted after dye conjugated with a caspase cleavage sequence is cleaved and binds to cell DNA; quantitation of green fluorescence counts at 24 h and 48 h for (**b**) K6L9(1X), (**c**) K6L9(4X), and (**d**) WTAS-K6L9(4X) was performed. All data were collected from three technical and biological replicates and recorded as mean ± SD. * = *p* < 0.05, ** = *p* < 0.01.

**Figure 4.**
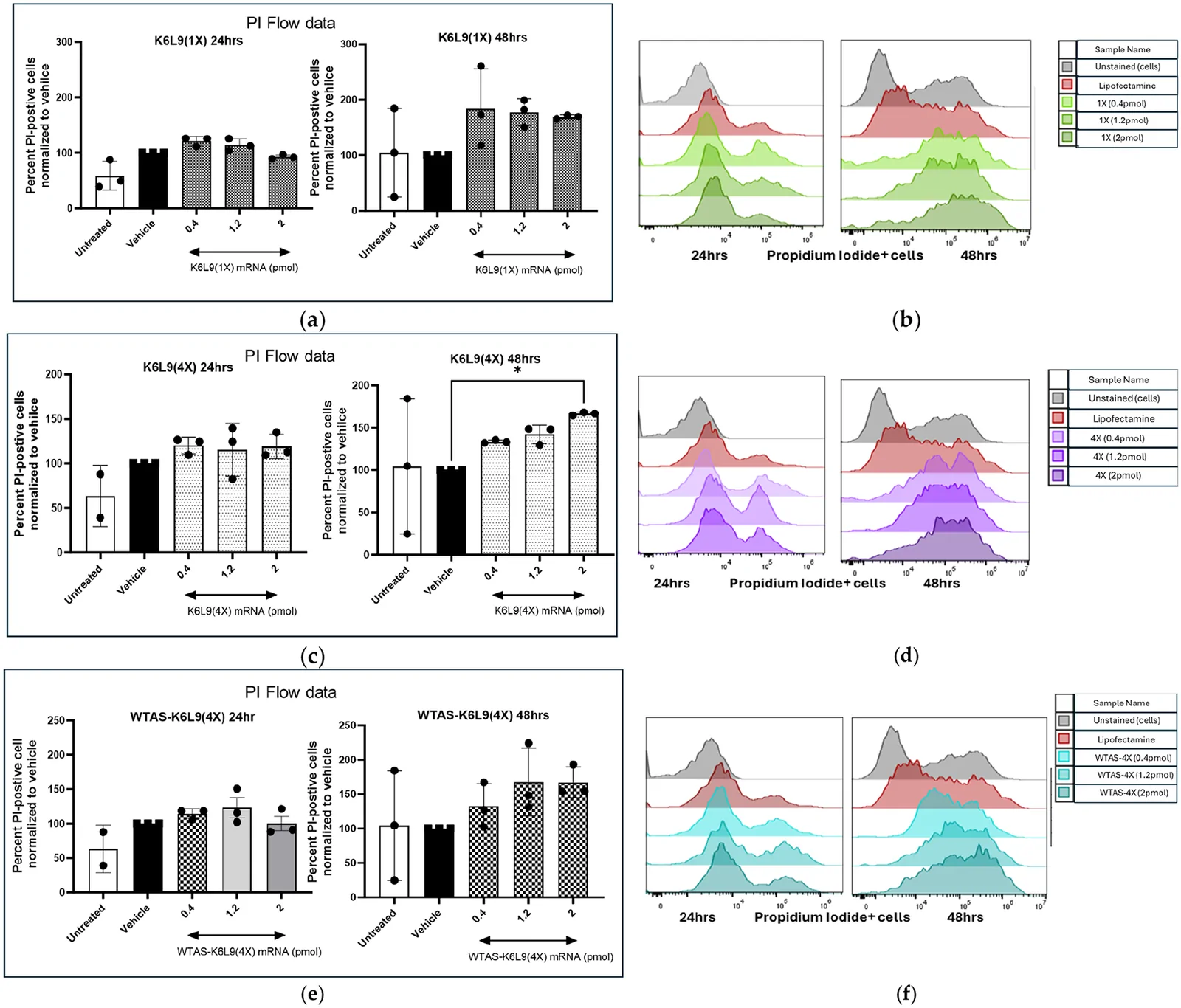
Cytotoxic effect of the translated peptide in 4T1 cells observed at 48 h. Flow cytometric analysis showing the percentage of PI-positive cells normalized to vehicle control at 24 and 48 h after transfection with (**a**) K6L9(1X) mRNA, (**c**) K6L9(4X) mRNA, and (**e**) WTAS-K6L9(4X) mRNA. Representative histograms of the PI-positive cells at 24 and 48 h post-transfection with (**b**) K6L9(1X), (**d**) K6L9(4X), and (**f**) WTAS-K6L9(4X) mRNAs are shown. The data were collected from three biological replicates and recorded as mean ± SD. * = *p* < 0.05.

**Figure 5.**
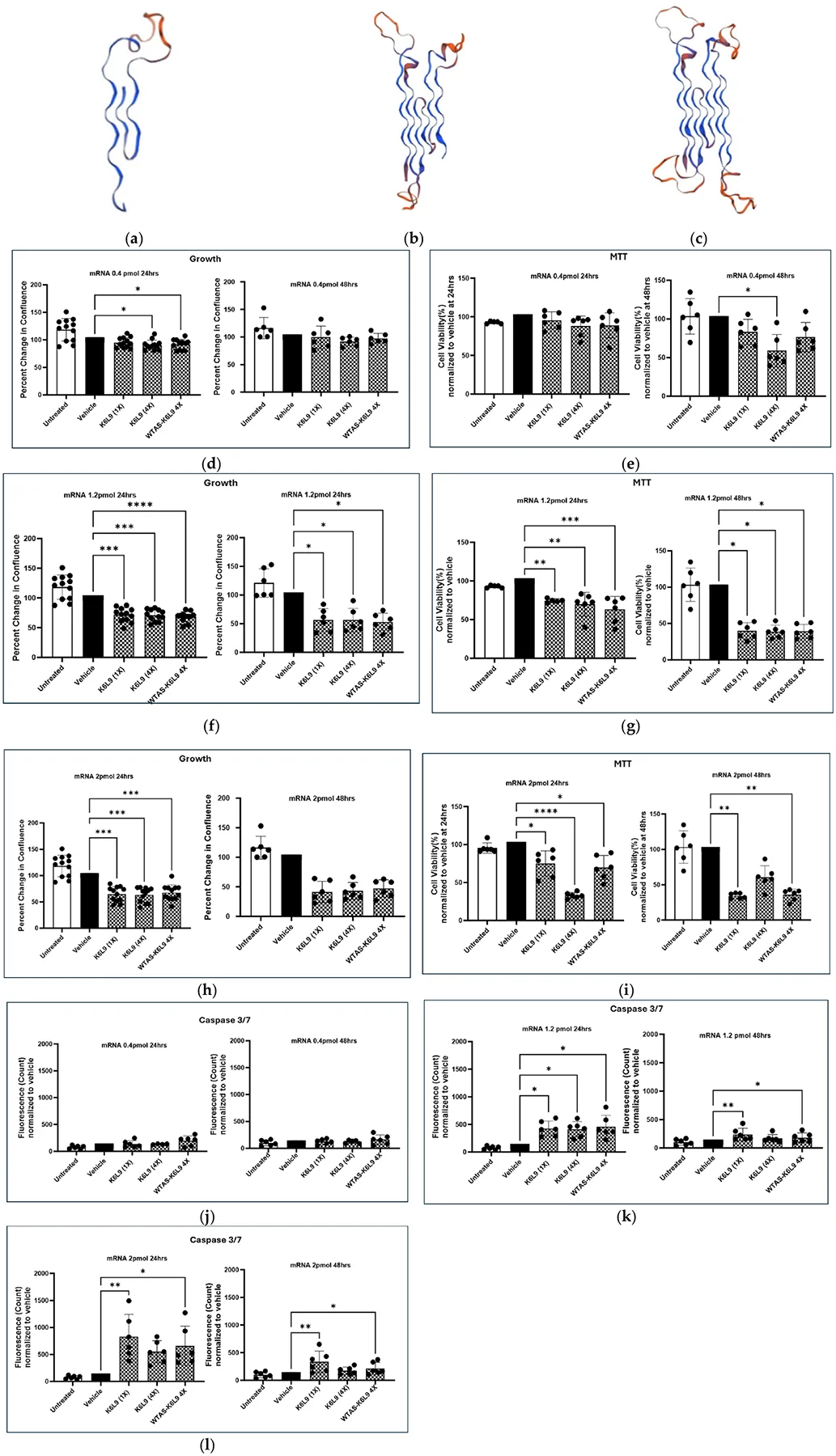
ACPs generated through mRNA transfection are similar in cytotoxic activity: SWISS-MODEL protein structures resulting from the mRNA design are shown for (**a**) K6L9(1X), (**b**) K6L9(4X), and (**c**) WTAS-K6L9(4X). 4T1 cells were transfected with K6L9(1X), K6L9(4X), and WTAS-K6L9(4X) mRNAs and compared at 3 different concentrations. Confluence was normalized and plotted as a percentage of the vehicle. Data are represented as change in confluence for (**d**) 0.4 pmol, (**f**) 1.2 pmol, and (**h**) 2 pmol. Cell viability was determined by MTT assay at 24 and 48 h after transfection of the mRNAs. Data are represented as percent viability normalized to the vehicle for (**e**) 0.4 pmol, (**g**) 1.2 pmol, and (**i**) 2 pmol. Quantitation of green fluorescence counts at 24 h and 48 h for (**j**) 0.4 pmol, (**k**) 1.2 pmol, and (**l**) 2 pmol was performed. The data were collected from three technical and experimental replicates and recorded as mean ± SD. * = *p* < 0.05, ** = *p* < 0.01, *** = *p* < 0.001, **** = *p* < 0.0001.

## Data Availability

The data will be available from Dr. Kamath upon request.

## Abbreviations

The following abbreviations are used in this manuscript:

IVT: In vitro transcription
mRNA: Messenger ribonucleic acid
MTT: 3-(4,5-Dimethylthiazol-2-yl)-2,5-diphenyltetrazolium bromide
ACP: Anticancer peptide
PI: Propidium iodide
K6L9: LKLLKKLLKKLLKLL amino acid sequence
D-K6L9: LKLLKKLLKKLLKLL
SA-K6L9-AS: SALKLLKKLLKKLLKLLAS amino acid sequence
K6L9(1X): Fusion peptide with one SA-K6L9-AS repeat
K6L9(4X): Fusion peptide with four SA-K6L9-AS repeats
WTAS-K6L9(4X): Fusion peptide with four SA-K6L9-AS repeats plus cell-penetrating peptide WTAS
WTAS-K6L9(4X)-GFP: Fusion peptide with four SA-K6L9-AS repeats plus cell-penetrating peptide WTAS plus GFP
GFP: Green fluorescent protein
FITC: Fluorescein isothiocyanate
NIR: Near-infrared
CDS: Coding sequence
AES: Amino-terminal enhancer of split
mtRNR1: Non-coding mitochondrial 12S rRNA
