# Supplementary material for: Augmenting the Cytotoxicity of Anticancer Peptide K6L9 by In Vitro-Synthesized mRNA
Source: Appl Sci (Basel). Author manuscript; Available in PMC 2026 Nov 6. (PMC13739426; doi:10.3390/app16073288)
Supplement: Supplementary file 1 [file NIHMS2214672-supplement-applsci-4165037-supplementary_tables_and_figures_A.pdf]

Figure S1: In vitro synthesis and validation of mRNAs; Figure S2: Expression of WTAS-K6L9(4X)-GFP mRNA in 4T1 cells induces cytotoxicity; Figure S3: Translation of K6L9 mRNAs had no significant effects in Annexin V binding or cell membrane integrity at 24- and 48-hours post transfection; Figure S4: Cytotoxic effect of the translated peptide observed at 48hrs; Figure S5: ACPs generated through mRNA transfection are similar in cytotoxic effect; Figure S6: Representative flow cytometry 4-quadrant graphs for ACPs generated through mRNA transfection. Table S1: DNA template sequences for K6L9(1X), K6L9(4X, WTAS-K6L9(4X) and WTAS-K6L9(4X)-GFP mRNA, AES-3′UTR, and mtRNR1-3′UTR; Video S1: Time lapse video of GFP expression in 4T1 cells transfected with GFP-WTAS-K6L9(4X).

Supplemental Table S1. DNA template sequences for CDS of K6L9(1X), K6L9(4X, WTAS-K6L9(4X) and WTAS-K6L9(4X)-GFP mRNA, AES-3′UTR, and mtRNR1-3′UTR.

mRNAs of interest	DNA CDS sequences for IVT mRNA generation
SA-K6L9-SA(1X)	5′-ATGGGCGGCAGCGGCGGCAGCGGCG GCAGCGGCGGCAGCGCGCT GAAACTGCTGAAAAAACTGCTGAAAAAACTGCTG AAACTGCTGGC G AGCGGC GGCAGCGG CGGCAGCGGCG GCAGCGGCGGC-3′
K6L9(4X)	5′-ATGGGCGGCAGCGGCGGCAGCGGC GGCAGCGGCGGCAGCGCGCT GAAACT GCTGAAAAAACTGCTGAAAAAACTGCT GAAACTGCTGGCG AGCGGCGGCAGCGG CGGCAGCGGCGGCAGCGGCGGCAGCGCGCTG AAACTGCTGAAAAAACTGCTGAAAAAACTGCTGAAACTGCTGGCGAG CGGCGGCAGCGGCGGCAGCGGCGGCAGCG GCGGCGGCGGCAGCGG CGGCAGCGGCG GCAGCGGCGGCAGCGCGCTGAAACTGCTGAAAAA ACTGCTGAAAAAACTGCTGAAACTGCTGGCGAGCGGCGGCAGCGGC GGCAGCGGCGGCAGCGGCGGCAGCGCG CTGAAACTGCTGAAAAAA CTGCTGAAAAAACTGCTGAAACTGCTGGCGAGCGGCGGCAGCGGCG GCAGCGGCGGCAGCGG CGGC-3′
WTAS-K6L9(4X)	5′-ATGCCCCTGAAGTGGCCTGGCAAGAAG AAGAAGGGCAAACCTGGAAAAAGAAA GGAACAGGAGAAGAAAAAGCGGAGAACCAGAGG CGGAAGCGGCGGCAGCGGC GGAAGCGGCGGCTCTGCCCTGAAACTG CTGAAGAAGCTGCTGAAAAAGCTGCTGAAGCTGCTGGCTAGCGGCGG CAGCGGCGGTTCCGGCGGCAGCGGCGGCAGCGCCCTGAAGCTGCTGA AGAAGCTCCTGAAG AAGCTGCTGAAGCTGCTGGCCAGCGGCGGCTC TGGCGGCTCCGGCGGCAGCGGC GGAGGCGGCTCTGGCGGCAGCGGC GGAAGCGGCGGCAGCGCCCTGAAGCTGCTGAAGAAGCTGCTGAAAA AGCTGCTGAAGCTGCTGGCCAGCGGAGGCAGCGGC GGCAGCGGAGGAAGCGGAGGCTCTG CTCTGAAGCTGCTCAAAAAGCTGCTGAAGAA ACTGCTTAAGCTGCTGGCCTCTGG CG GCTCCGGCGGCAGCGGCGGA TCCGG CGGA-3′
WTAS-K6L9(4X)-GFP	5′- ATGCTGAAGTGGCCCGGCAAGAAGAAGAAGGGCAAGCCTGGCAAGC GCAAAGAGCAAGAGAAGAAAAAGCGGCGGACCAGAGG CGGCTCTGGCGGAAGCGGAGGATCTGGTGGATCTGCCCTGAAGCT GCTGAAGAAACTGCTGAAAAAGCTCCTCAAGCTGCTGGCCA G TG GTGGCAGCGGTGGTAGCGGAGGTAGTGGCGGATCTGCTCTCAAAC TC CTCAAGAAGTTGCTCAAGAAGCTCCTGAAACTGCTCGCCTCA GGC GGAAGTGGTGGTTCAGGCGGATCCGGCGGAATGAGAAAGG GCGAAGAACTGTTCACCGGCGTGGTGCCCATTCTGGTGGAACTGGAC GGGGATGTGAACGGCCACAAGTTTAGCGTTAGCGGCGAAG GCGAAGGGGATGCCACAAACGGAAAGCTGACCCTGAAGTTCATC TGCACCACCGGCAAGCTGCCTGTGCCTTGGCCTACACTGGTCACCACA

	CTGACATACGGCGTGCAGTGCTTCGCCAGATATCCCGACCA TATGAAGCAGCACGACTTCTTCAAGAGCGCCATGCCTGAGGGCTA CGTGCAAGAGAGAACCATCAGCTTCAAGGACGACGGCACCTAC AAGACCAGAGCCGAAGTGAAGTTCGAGGGCGACACCCTGGTCAA CCGGATCGAGCTGAAGGGCATCGACTTCAAAGAGGACGGCAACA TCCTGGGCCACAAGCTCGAGTACAACTTCAACAGCCACAACGTGTAC ATCACCGCCGACAAGCAGAAGAACGGCATCAAGGCCAACT TCAAGATCCGGCACAACGTGGAAGATGGCAGCGTGCAGCTGGCC G ATCACTACCAGCAGAATACCCCTATCGGCGACGGACCTGTGCT GCTGCCCGATAATCACTACCTGAGCACACAGAGCGCCCTGAGCA AGGACCCCAACGAGAAGAGGGATCACATGGTGCTGCTGGAATTC GTGACCGCCGCTGGCATCACACACGGCATGGATGAGCTGTACA AACGGCCTGCCGCCAACGACGAGAATTATGCCGCTTCTGTTGGAG GCAGCGGAGGAAGCGGTGGCTCCGGCGGTAGTGCTCTGAAGCTC CTTAAAAAGCTTCTCAAAAAGCTGCTTAAGCTCCTCGCTTCTGGC GGCAGTGGCGGTAGCGGTGGTTCTGGCGGTTCTGCATTGAAACTG CTTAAGAAACTTCTCAAGAAACTGTTGAAGCTGCTTGCCAGCGGA GGTTCCGGCGGCTCAGGTGGTAGCGGTG -3′
AES–3′UTR	5′- CTGGTACTGCATGCACGCAATGCTAGCTGCCCCTTTCCCGTCCT GGGTACCCCGAGTCTCCCCCGACCTCGGGTCCCAGGTATGCTCCC ACCTCCACCTGCCCCACTCACCACCTCTGCTAGTTCCAGACACCTC C -3′
mtRNR1–3′UTR	5′- CAAGCACGCAGCAATGCAGCTCAAAACGCTTAGCCTAGCCAC ACCCCCACGGGAAACAGCAGTGATTAACCTTTAGCAATAAACGA AAGTTTAACTAAGCTATACTAACCCCAGGGTTGGTCAATTTCGTG CCAGCCACACC -3′

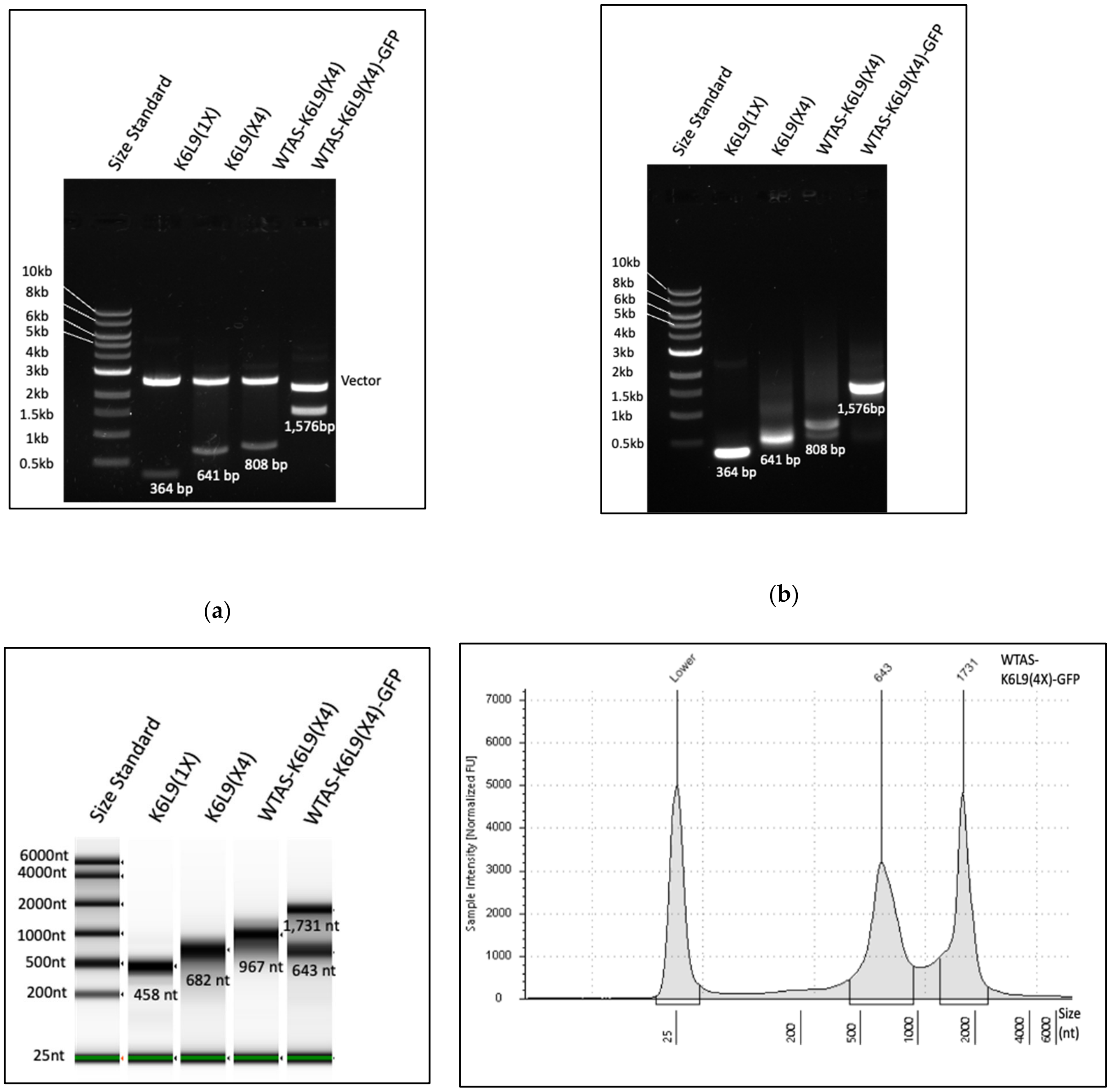

(c) (d)

Figure S1. In vitro synthesis and validation of mRNAs: (**a**) Agarose gel image showing restriction digestion of the cloning plasmids carrying K6L9(1X), K6L9(4X), WTAS-K6L9(4X) (from Figure 1), and WTAS-K6L9(4X)-GFP template DNA insert. Size standard is indicated in kilobase pairs (kb) while band sizes are indicated in base pair (bp). Vector refers to the plasmid backbone; (**b**) Agarose gel image showing PCR amplified K6L9(1X), K6L9(4X), WTAS-K6L9(4X), (from Figure 1), and WTAS-K6L9(4X)-GFP template DNA. Size standard is indicated in kilobase pairs (kb) while band sizes are indicated in base pair (bp); (**c**) RNA Screen tape image showing IVT synthesized mRNA bands of K6L9(1X), K6L9(4X), WTAS-K6L9(4X) (From Figure 1), and WTAS-K6L9(4X)-GFP. Size standard and band sizes are indicated in nucleotides (nt); (**d**) RNA Screen Tape assay electropherogram showing the WTAS-K6L9(4X)-GFP mRNA size in nt.

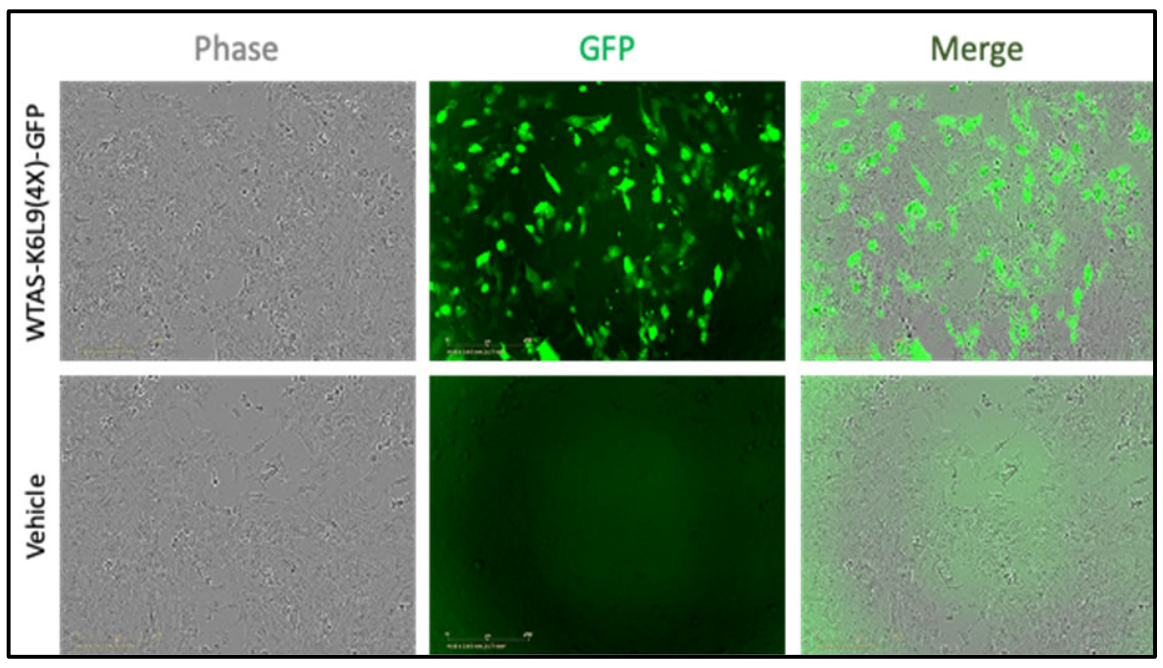

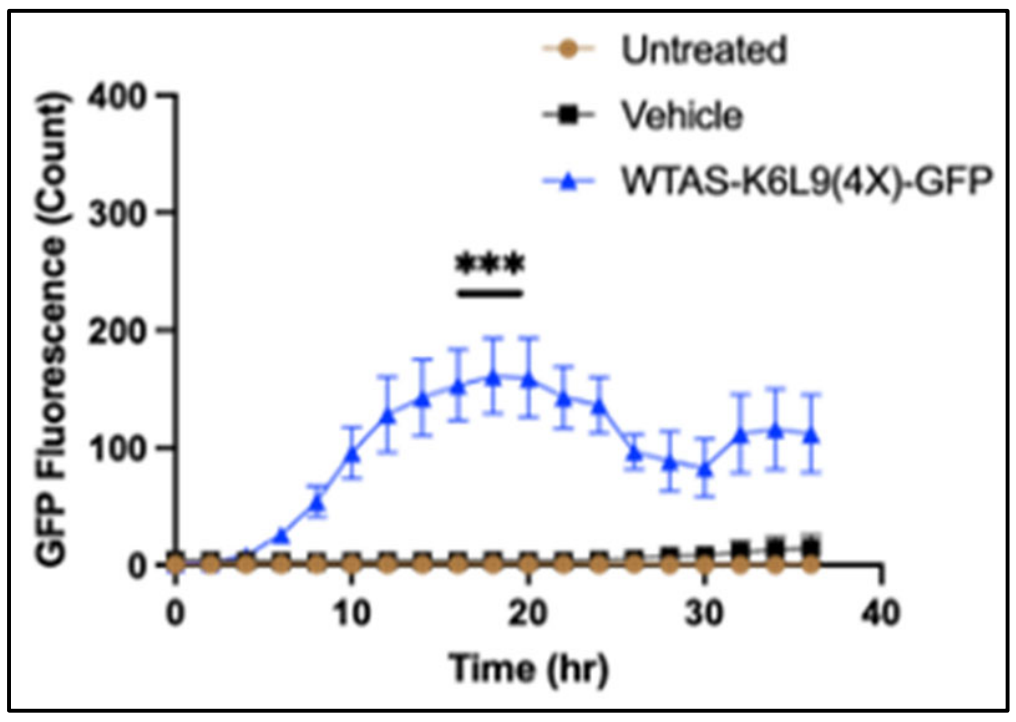

(a)

(b)

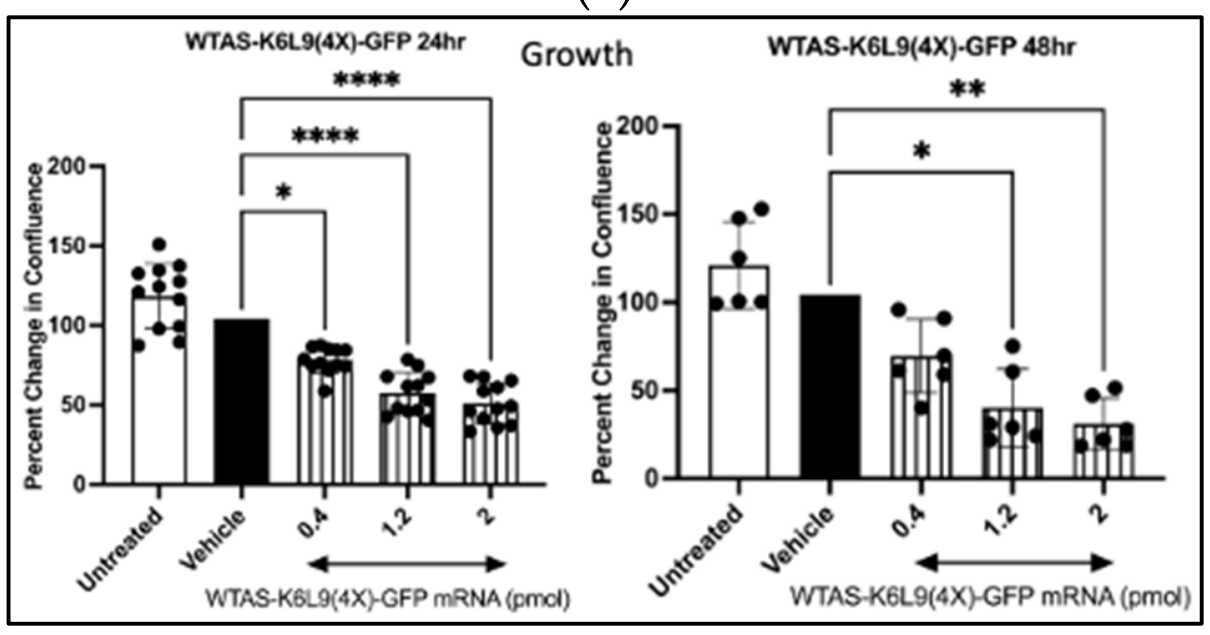

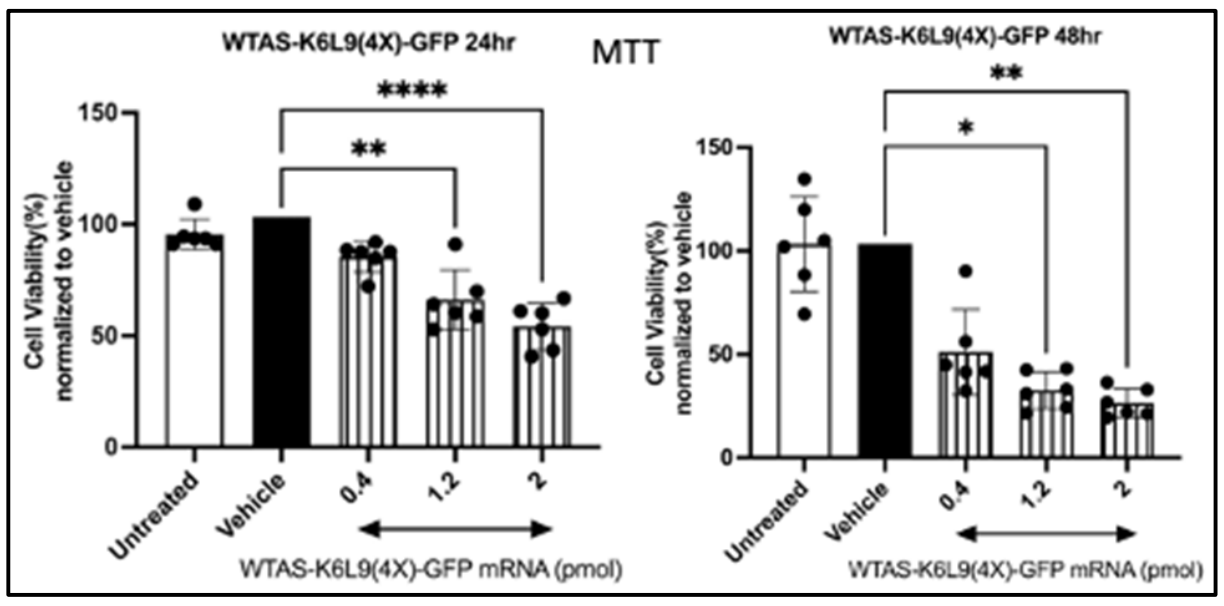

(c)

(d)

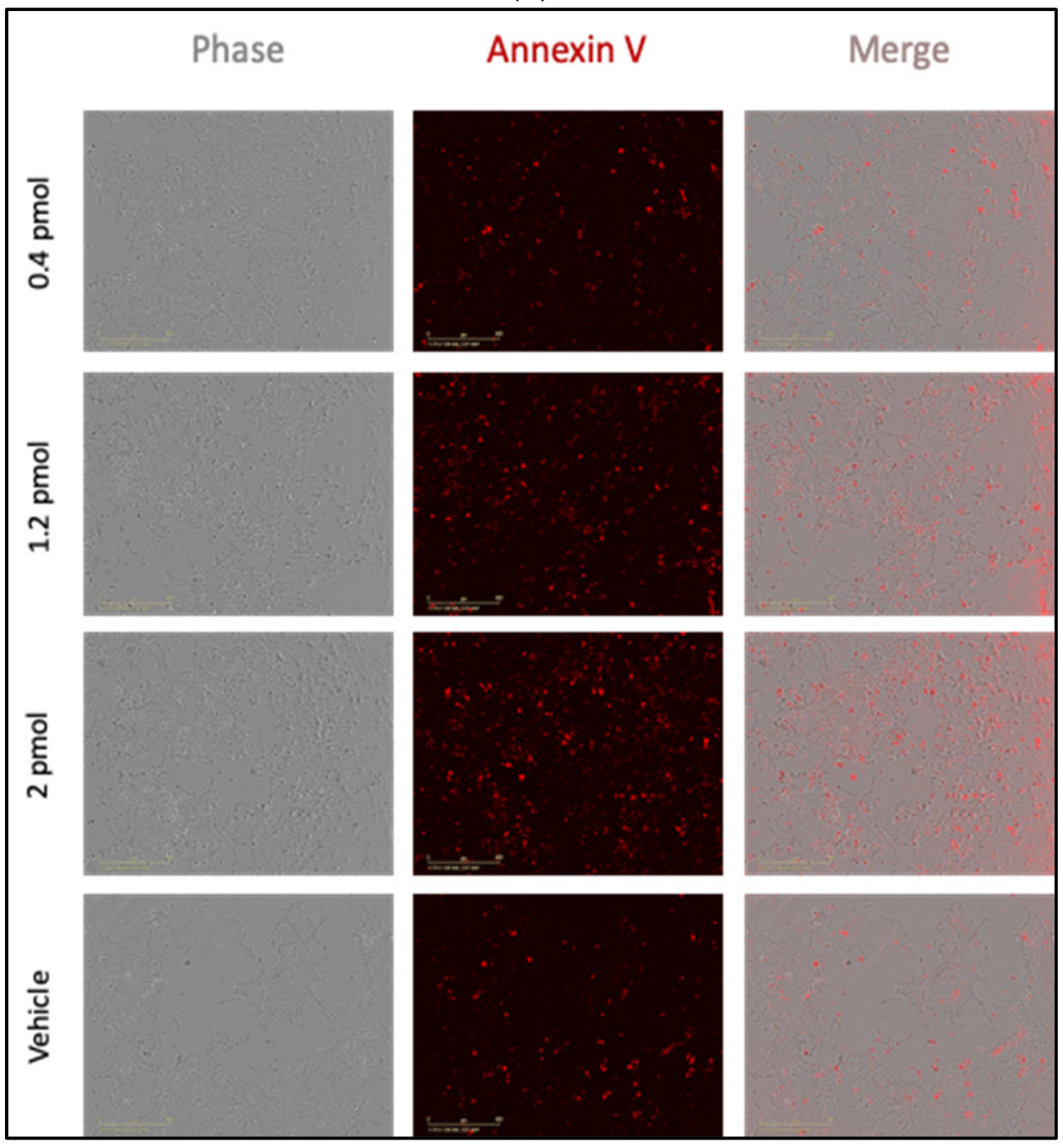

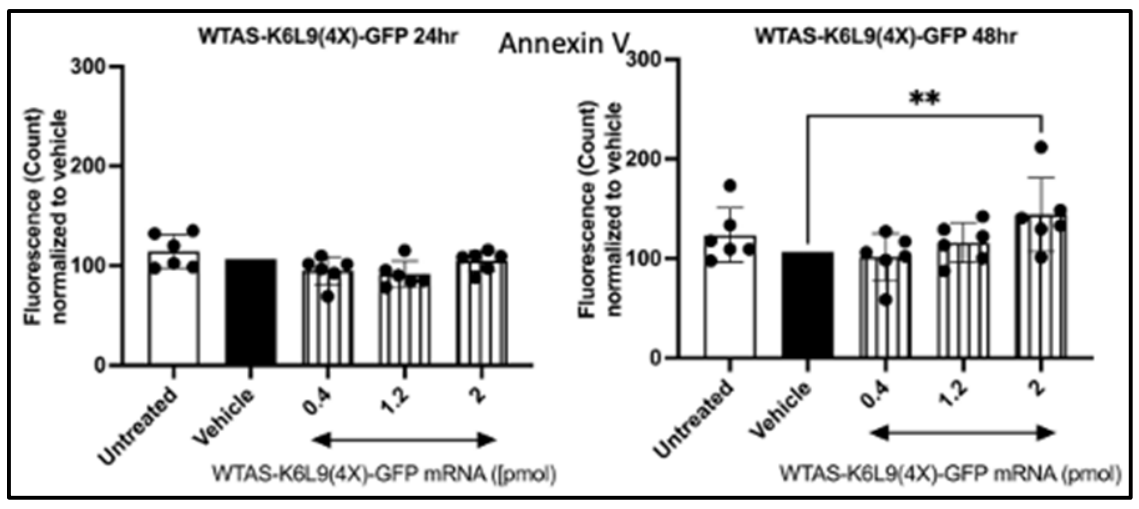

(e)

(f)

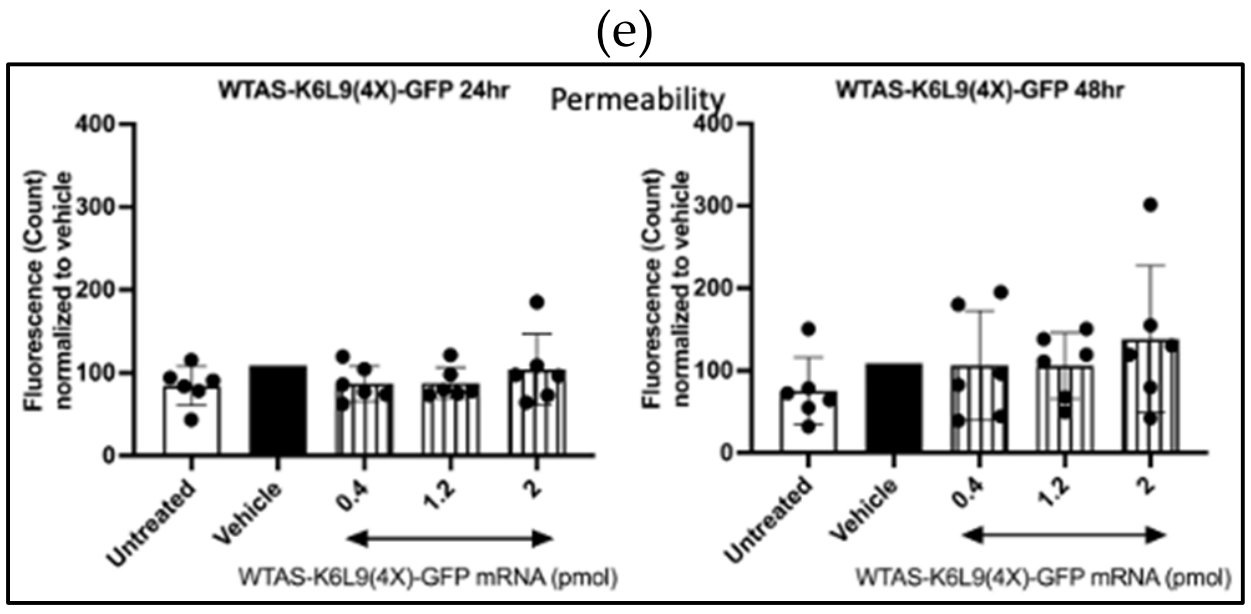

(g)

Figure S2. Expression of WTAS-K6L9(4X)-GFP mRNA 4T1 cells induces cytotoxicity: (**a**) Images showing expression of GFP after 4T1 cells expressed WTAS-K6L9(4X)-GFP mRNA at 2 pmol. The images were taken with a 10X objective at 24 hours; (**b**) Quantification of

GFP expression over 36 hours period. The data was collected every 2 hours using IncuCyte ®; (c) Cell growth measured by % total area covered by cells (confluence) at 24 and 48 hours after mRNA transfection. The cells were transfected with three mRNA concentrations 0.4, 1.2, and 2 pmol. Test confluence is normalized and plotted as a percentage to vehicle; **(d)** Cell viability determined by MTT assay at 24 and 48 hours after translation of WTAS-K6L9(4X)-GFP mRNA. Viability is expressed as a percentage normalized to the vehicle; (**e**) Images showing Annexin V binding after transfecting the 4T1 cells with WTAS-K6L9(4X)-GFP mRNA at 48 hours; (f) Quantitation of orange fluorescence counts in images in panel (e) after 24 and 48 hours; **(g)** DNA dye movement across damaged cell membrane after WTAS-K6L9(4X)-GFP mRNA translation for 24 and 48 hours. The dye binds the DNA with increase in fluorescence; The data was collected from three technical and biological replicates and recorded as mean±SD. *= p<0.05, ** = p<0.01, ***=p < 0.001. *** = p< 0.001, **** p< 0.0001

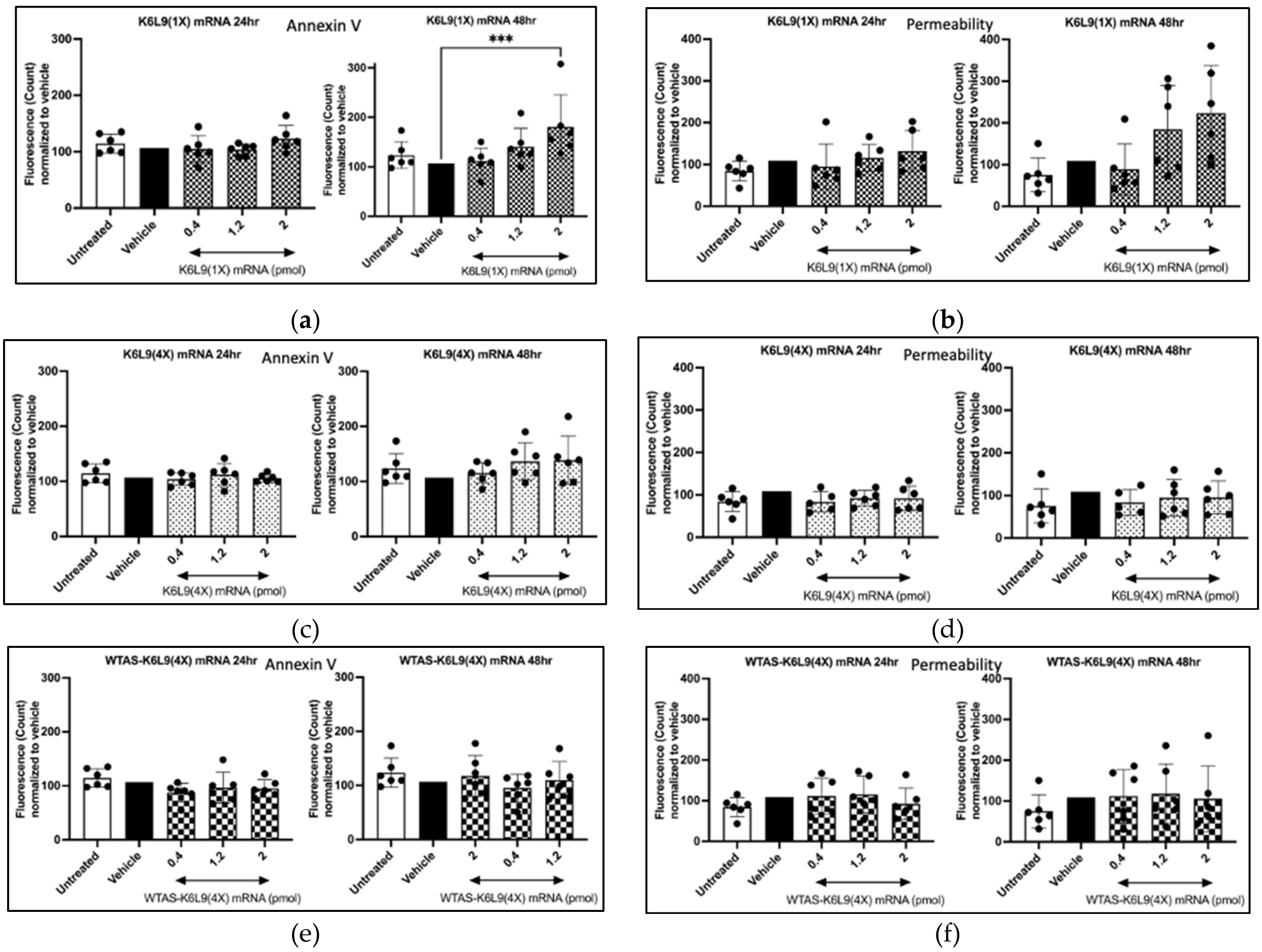

Figure S3. Translation of K6L9 mRNAs had no significant effects in Annexin V binding or cell membrane integrity at 24- and 48-hours post transfection: (**a**) Annexin V binding after K6L9(1X) mRNA translation for 24 and 48 hours. Fluorescence is expressed as a percentage normalized to the vehicle; (**b**) DNA dye movement across damaged cell membrane after K6L9(1X) mRNA translation for 24 and 48 hours. The dye binds the DNA with increase in fluorescence; (c) Annexin V binding after K6L9(4X) mRNA translation for 24 and 48 hours; (d) DNA dye movement across damaged cell membrane after K6L9(4X) mRNA translation for 24 and 48 hours; (**e**) Annexin V binding after WTAS-K6L9(4X) mRNA translation for 24 and 48 hours; **(f)** DNA dye movement across damaged cell membrane after WTAS-K6L9(4X) mRNA translation for 24 and 48 hours. The data was collected from three technical and biological replicates and recorded as mean ± SD. *= p<0.05, ** = p<0.01, ***=p < 0.001. *** = p< 0.001, **** p< 0.0001.

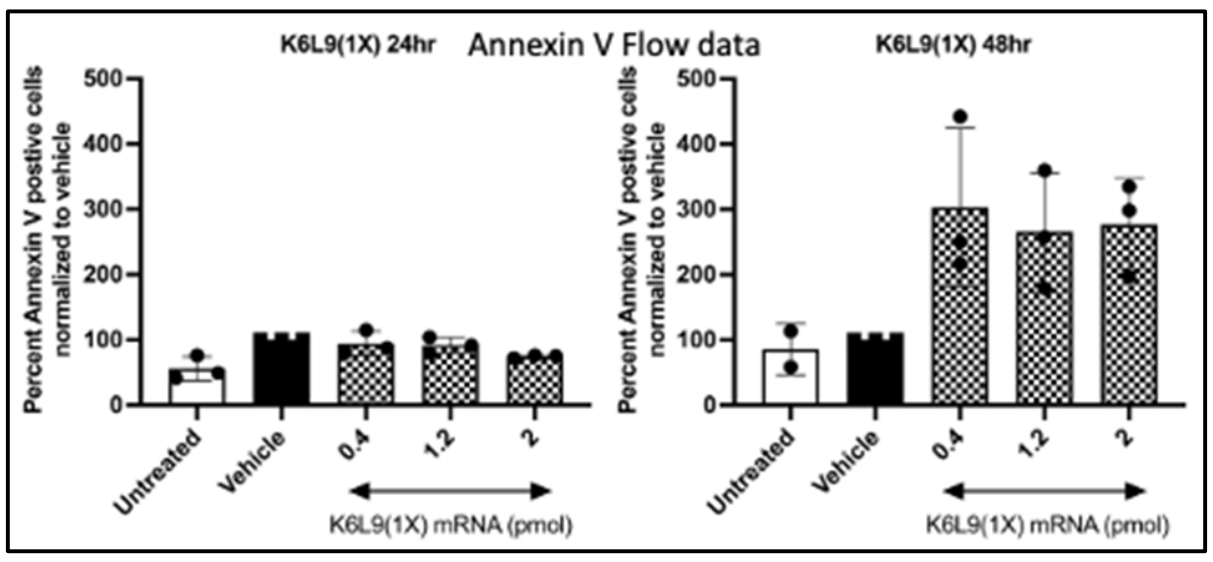

(a)

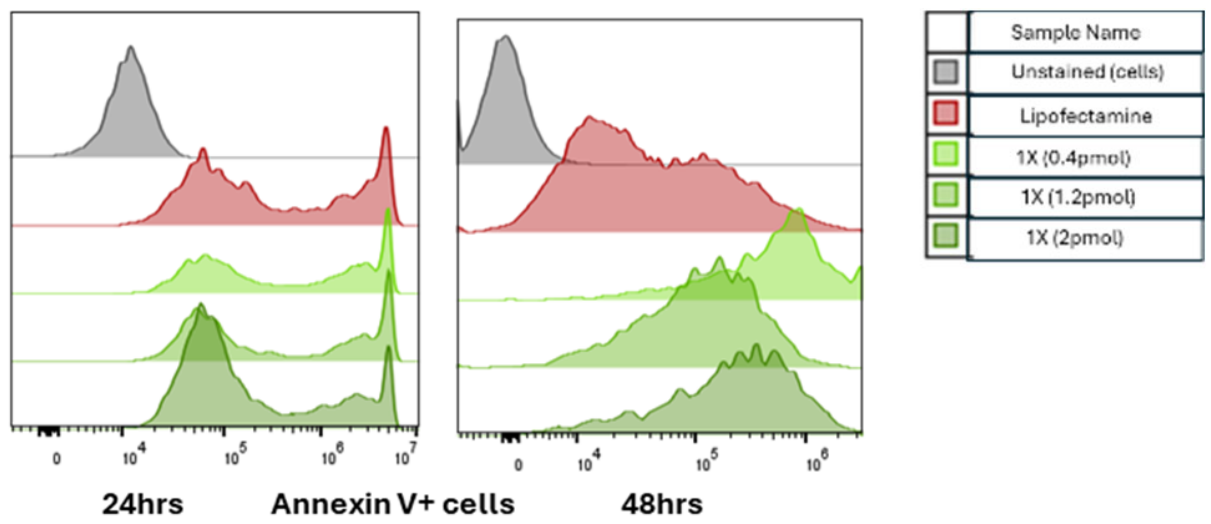

(b)

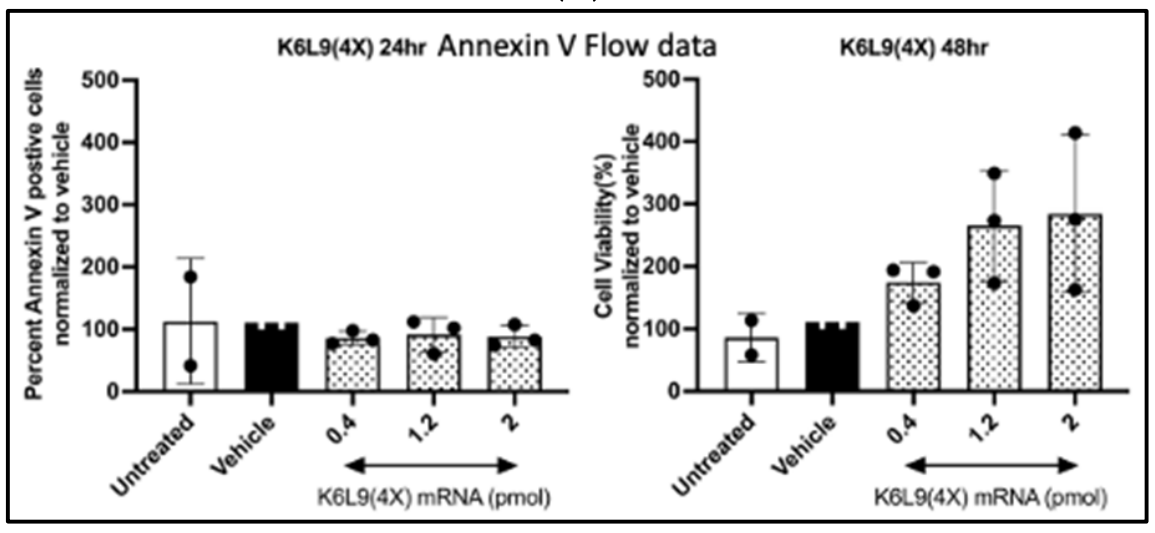

(c)

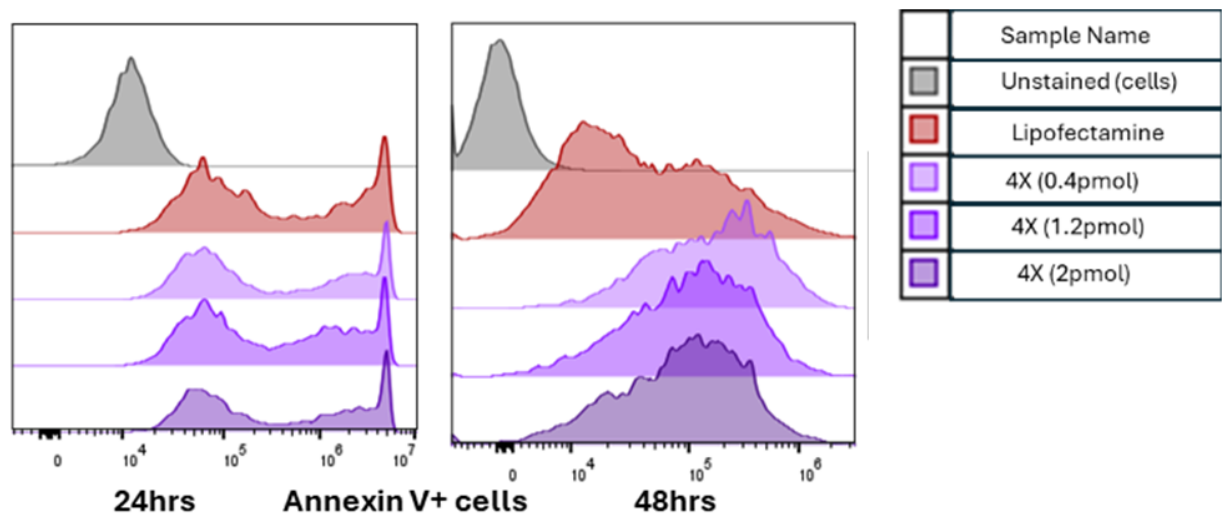

(d)

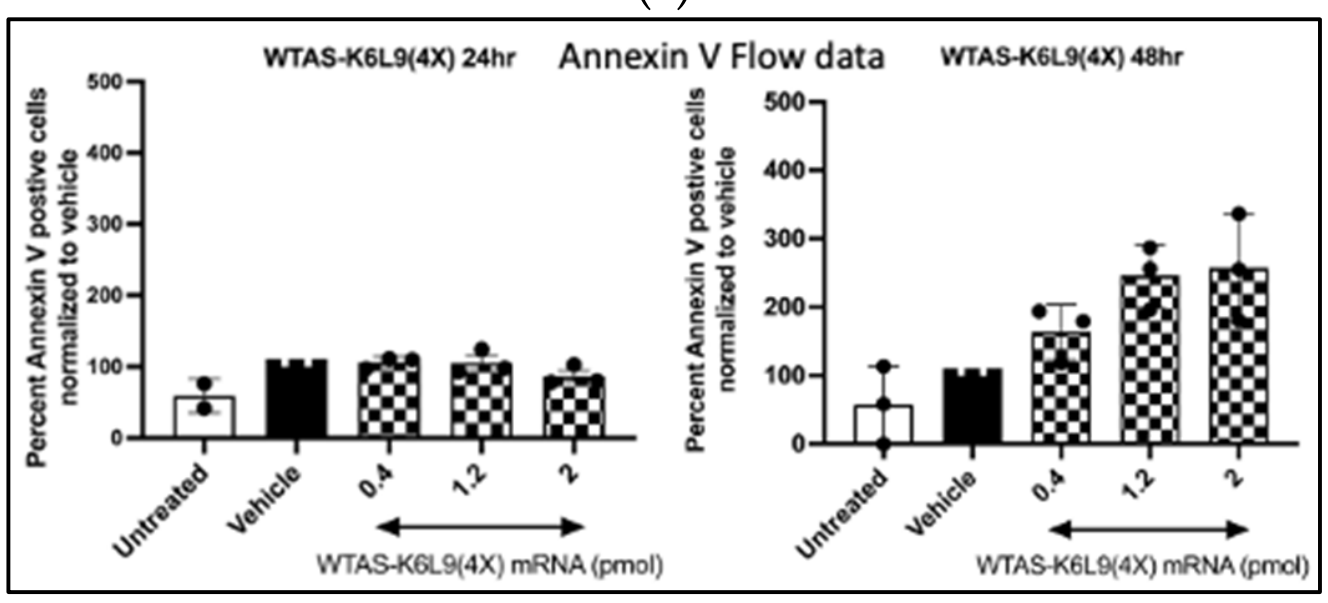

(e)

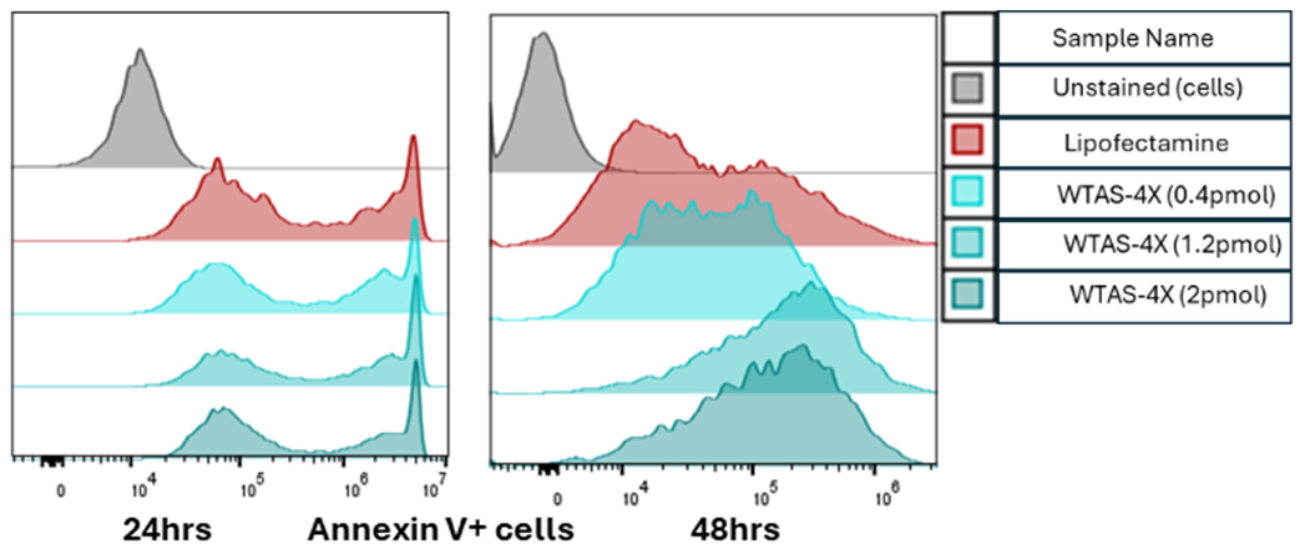

(f)

Figure S4. Cytotoxic effect of the translated peptide observed at 48hrs: Flow cytometric analysis showing the percentage of Annexin V positive cells normalized to vehicle control at 24 and 48 hours after transfection with (a) K6L9(1X) mRNA; (**c**) K6L9(4X) mRNA; (e) WTAS-K6L9(4X) mRNA. Representative histograms of Annexin V positive cells at 24 and 48hrs post-transfection with (b) K6L9 (1X); (d) K6L9 (4X) and (f) WTAS-K6L9 (4X) mRNAs are shown. The data were collected from three biological replicates and recorded as mean±SD. *= $p<0.05$.

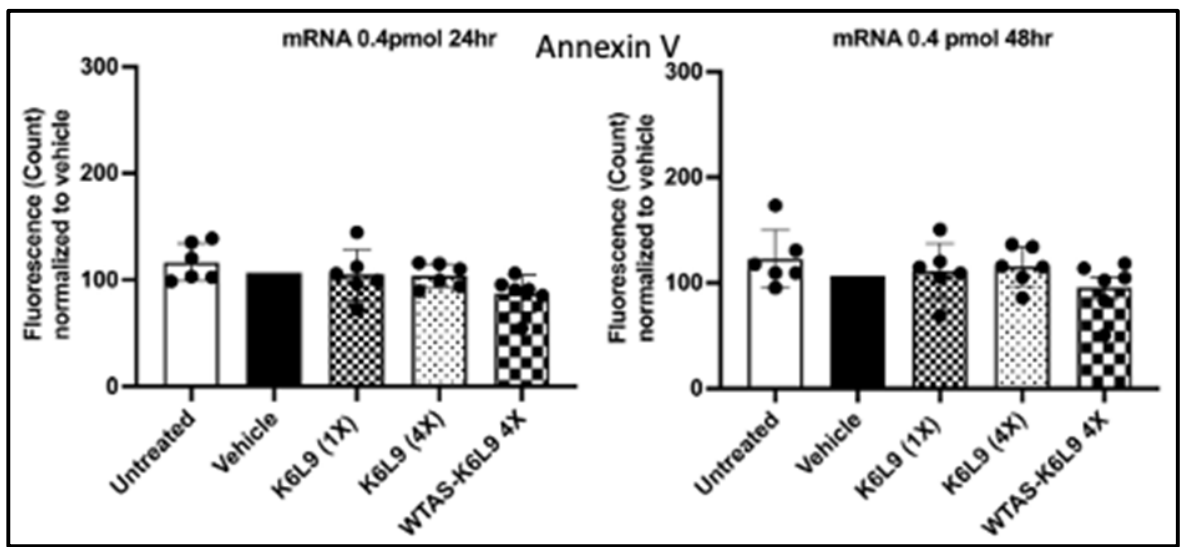

(**a**)

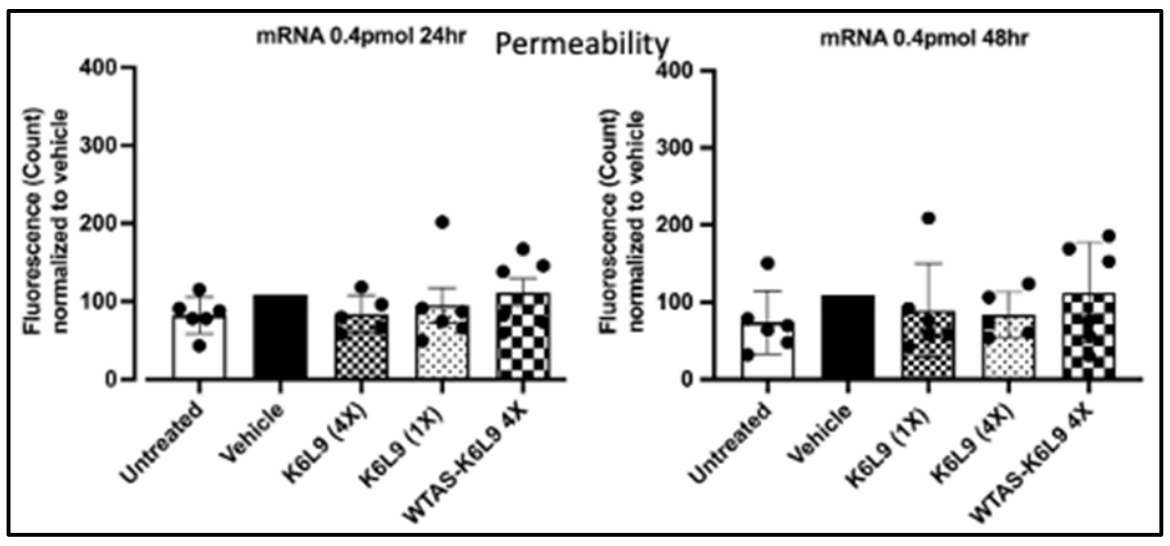

(**b**)

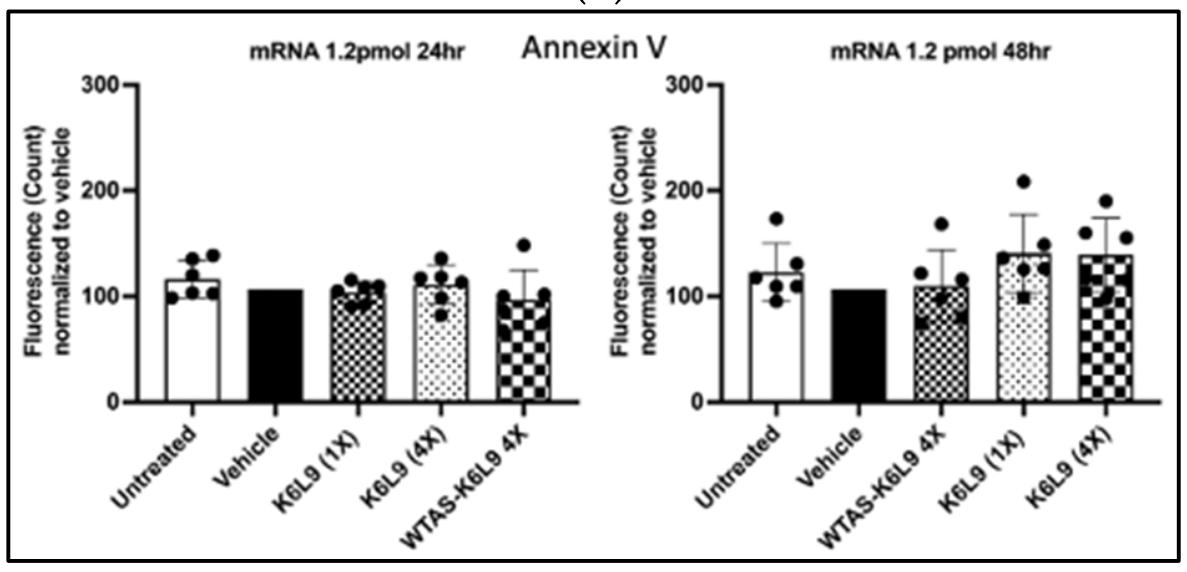

(c)

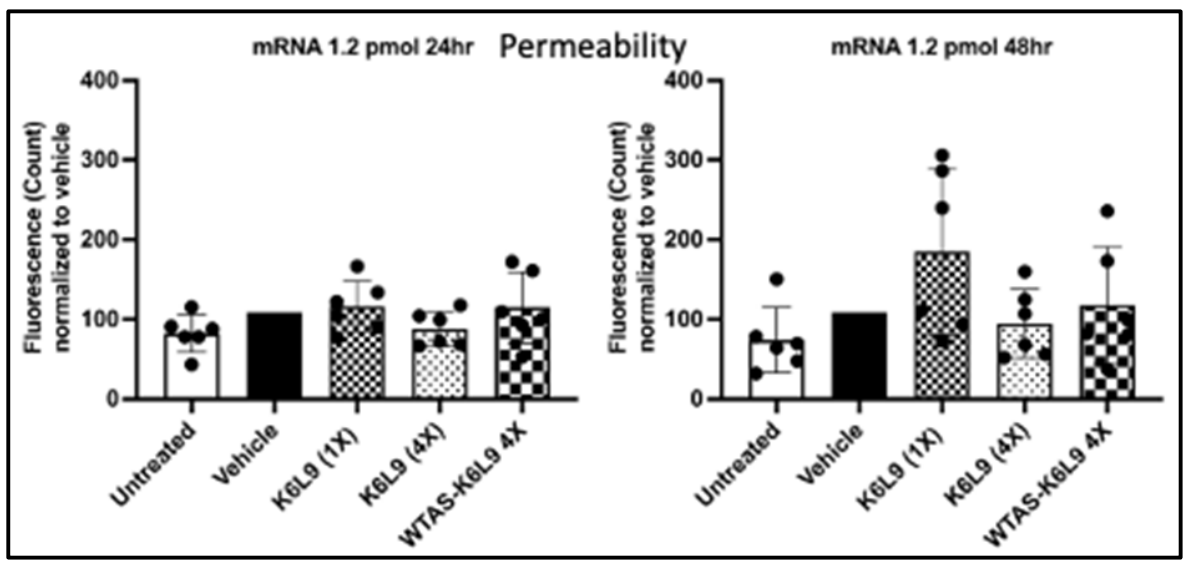

(d)

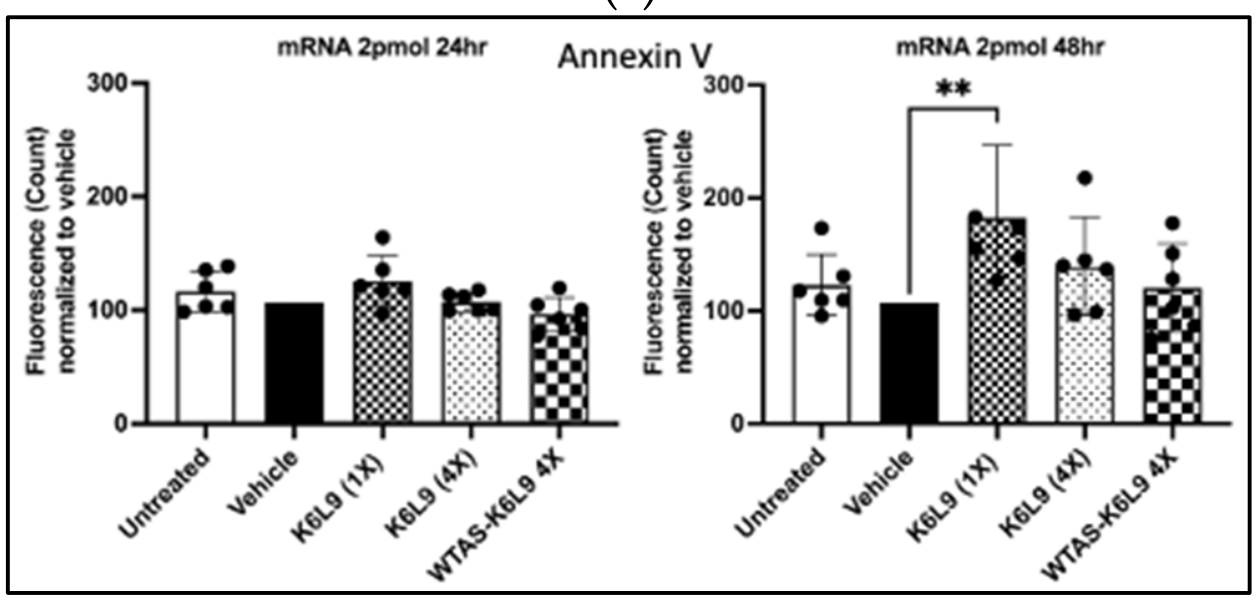

(**e**)

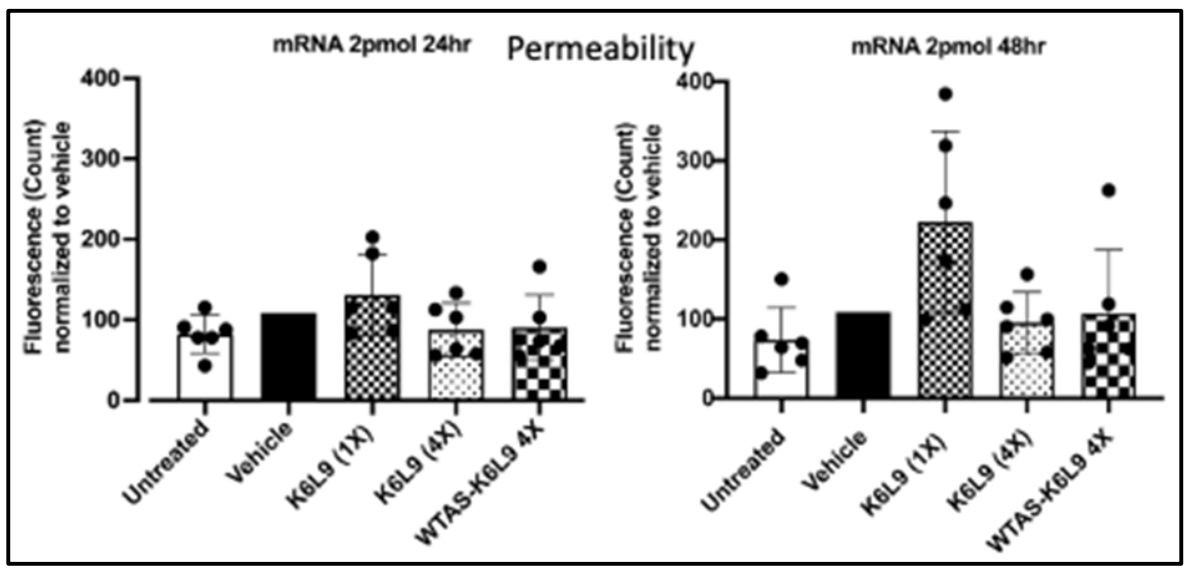

(f)

Figure S5. ACPs generated through mRNA transfection are similar in cytotoxic activity: 4T1 cells were transfected with K6L9 (1X), K6L9 (4X) and WTAS-K6L9 (4X) mRNAs and compared at 3 different concentrations. (**a**) Annexin V binding after 0.4pmol mRNA translation for 24 and 48 hours. Fluorescence is expressed as a percentage normalized to the vehicle; (**b**) DNA dye movement across damaged cell membrane after 0.4pmol mRNA translation for 24 and 48 hours. The dye binds the DNA with increase in fluorescence; (c) Annexin V binding after 1.2pmol mRNA translation for 24 and 48 hours; (d) DNA dye movement across damaged cell membrane after 1.2pmol mRNA translation for 24 and 48 hours; (**e**) Annexin V binding after 2pmol mRNA translation for 24 and 48 hours; **(f)** DNA dye movement across damaged cell membrane after 2pmol mRNA translation for 24 and 48 hours. The data was collected from three technical and biological replicates and recorded as mean ± SD. *= p<0.05, ** = p<0.01, ***=p < 0.001. *** = p< 0.001, **** p< 0.0001

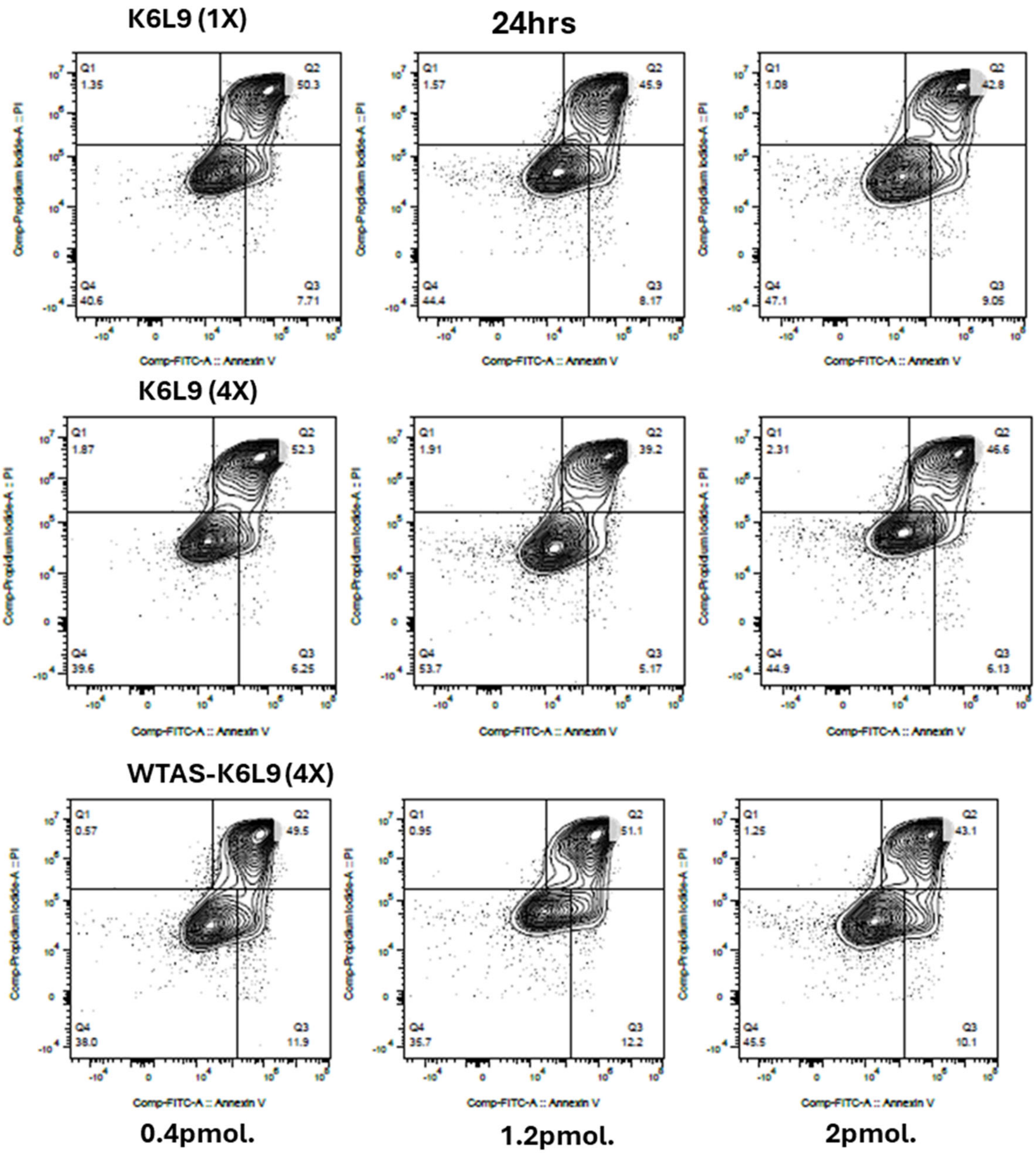
K6L9 (1X)
24hrs
Q1 1.35
Q2 50.3
Q4 40.6
Q3 7.71
Q1 1.57
Q2 45.9
Q4 44.4
Q3 8.17
Q1 1.08
Q2 42.8
Q4 47.1
Q3 9.05
K6L9 (4X)
Q1 1.87
Q2 52.3
Q4 39.6
Q3 6.25
Q1 1.91
Q2 39.2
Q4 53.7
Q3 5.17
Q1 2.31
Q2 46.6
Q4 44.9
Q3 6.13
WTAS-K6L9 (4X)
Q1 0.57
Q2 49.5
Q4 38.0
Q3 11.9
Q1 0.95
Q2 51.1
Q4 35.7
Q3 12.2
Q1 1.25
Q2 43.1
Q4 45.5
Q3 10.1
Comp-Propidium Iodide-A :: PI
Comp-FITC-A :: Annexin V
0.4pmol.
1.2pmol.
2pmol.

(a)

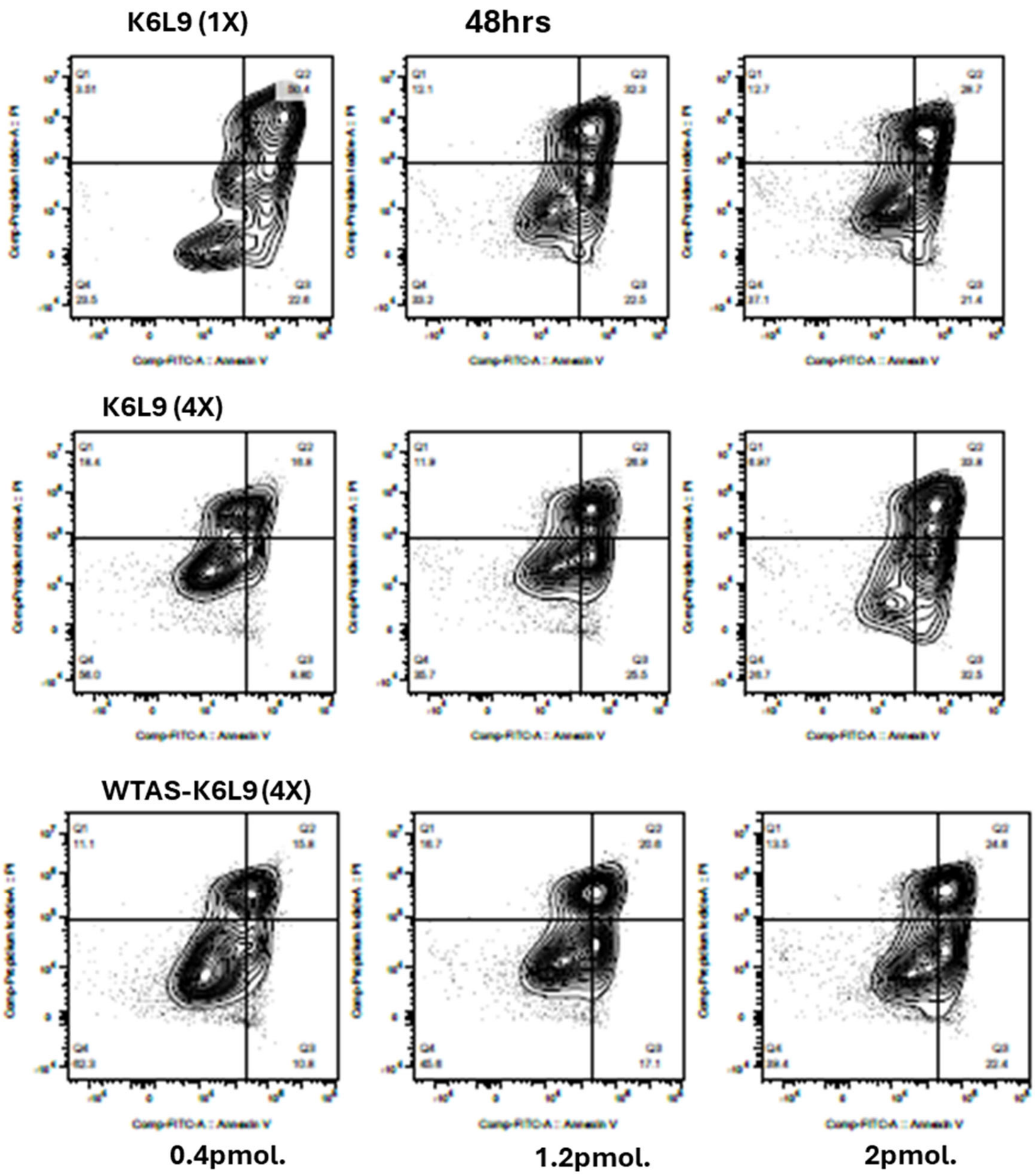

(b)

Figure S6. Representative flow cytometry 4-quadrant graphs for ACPs generated through mRNA transfection 4T1 cells were transfected with K6L9 (1X), K6L9 (4X) and WTAS-K6L9 (4X) mRNAs and compared at 3 different concentrations. (**a**) representative graph for all 3 peptides across 3 different concentrations at 24hrs. (b) representative graph for all 3 peptides across 3 different concentrations at 48hrs.
